# Short-term outcomes of robot-assisted versus conventional minimally invasive esophagectomy for esophageal cancer: a systematic review and meta-analysis of 18,187 patients

**DOI:** 10.1007/s11701-024-01880-3

**Published:** 2024-03-16

**Authors:** Rui Perry, José Pedro Barbosa, Isabel Perry, José Barbosa

**Affiliations:** 1 Faculty of Medicine, University of Porto, Porto, Portugal; 2 Department of Community Medicine, Information and Decision in Health, Faculty of Medicine, University of Porto, Porto, Portugal; 3 Department of Stomatology, São João University Hospital Center, Porto, Portugal; 4 Faculty of Medicine, University of Salamanca, Salamanca, Spain; 5 Department of Surgery and Physiology, Faculty of Medicine, University of Porto, Porto, Portugal; 6 Department of General Surgery, São João University Hospital Center, Porto, Portugal

**Keywords:** Esophagectomy, Esophageal cancer, Robotic surgery, Minimally invasive surgery, Short-term outcomes

## Abstract

The role of robotic surgery in the curative-intent treatment of esophageal cancer patients is yet to be defined. To compare short-term outcomes between conventional minimally invasive (cMIE) and robot-assisted minimally invasive esophagectomy (RAMIE) in esophageal cancer patients. PubMed, Web of Science and Cochrane Library were systematically searched. The included studies compared short-term outcomes between cMIE and RAMIE. Individual risk of bias was calculated using the MINORS and RoB2 scales. There were no statistically significant differences between RAMIE and cMIE regarding conversion to open procedure, mean number of harvested lymph nodes in the mediastinum, abdomen and along the right recurrent laryngeal nerve (RLN), 30- and 90-day mortality rates, chyle leakage, RLN palsy as well as cardiac and infectious complication rates. Estimated blood loss (MD − 71.78 mL, *p* < 0.00001), total number of harvested lymph nodes (MD 2.18 nodes, *p* < 0.0001) and along the left RLN (MD 0.73 nodes, *p* = 0.03), pulmonary complications (RR 0.70, *p* = 0.001) and length of hospital stay (MD − 3.03 days, *p* < 0.0001) are outcomes that favored RAMIE. A significantly shorter operating time (MD 29.01 min, *p* = 0.004) and a lower rate of anastomotic leakage (RR 1.23, *p* = 0.0005) were seen in cMIE. RAMIE has indicated to be a safe and feasible alternative to cMIE, with a tendency towards superiority in blood loss, lymph node yield, pulmonary complications and length of hospital stay. There was significant heterogeneity among studies for some of the outcomes measured. Further studies are necessary to confirm these results and overcome current limitations.

**Supplementary Information:**

The online version contains supplementary material available at 10.1007/s11701-024-01880-3.

## Introduction

Esophageal cancer is currently ranked seventh, globally, in terms of incidence, among cancer cases. It is also the sixth leading cause of cancer-related deaths, being responsible for 1 in every 18 cancer deaths in 2020. Histologically, the incidence of squamous cell carcinoma (SCC) has been in constant decline due to a change in economic gains, dietary regimes, and tobacco consumption—all major risk factors for esophageal cancer. Therefore, we are witnessing a shift in the histopathological subtypes, leading to an increase in the incidence of esophageal adenocarcinoma (AC) worldwide [1].

In spite of poor prognosis and long-term survival, the milestone of the disease’s primary management is currently set at radical esophagectomy and extended lymphadenectomy with previous eventual neoadjuvant chemoradiotherapy [2–6]. Its surgical approach underwent a paradigm change from open thoracotomy and laparotomy to conventional minimally invasive esophagectomy (cMIE), combining both thoracoscopy and laparoscopy [3]. This technique offers a decrease in total complication rates, wound infection rates, intraoperative blood loss, length of hospital stay, resulting in an improvement in both morbidity and mortality postoperatively [7–11]. Nonetheless, cMIE is not without limitations. Restricted movement of instruments, decreasing dexterity, prove to be technically complex and demanding, requiring a high number of patients to complete a surgeon’s learning curve [12, 13].

Robot-assisted minimally invasive esophagectomy (RAMIE) was first introduced in 2003 [14] and has since gradually proved to overcome some of cMIE’s limitations. Accounting for its articulated instruments, with 7 degrees of freedom, tremor filtering systems and improved magnification, precise dissection along narrower spaces is made simpler to surgeons [15, 16]. Whether these advantages represent better short- and long-term outcomes to esophageal cancer patients is still unclear.

Recently, several systematic reviews and meta-analyses reported RAMIE to be a safe and feasible alternative to cMIE in the treatment of esophageal cancer patients. RAMIE was associated with a tendency of longer operating time, less estimated blood loss and shorter length of hospital stay. RAMIE also yielded a larger number of lymph nodes and had lower rates of pulmonary complications when compared to cMIE [17–24].

Therefore, with the aim of analyzing RAMIE’s current contribution to the surgical approach of esophageal cancer patients, we conducted this systematic review and meta-analysis, assessing recent observational clinical studies (OCS) and randomized controlled trials (RCT), comparing short-term outcomes associated with RAMIE and cMIE. As robotic systems become more and more available worldwide and surgeons adhere to these, their technique upgrades over time. It becomes imperative to analyze the most recent data regarding this topic, as it is in constant evolution and rapidly changes as systems become more complex.

## Materials and methods

This systematic review and meta-analysis followed the PRISMA (Preferred Reporting Items for Systematic Reviews and Meta-Analyses) Statement guidelines and was prospectively registered on PROSPERO (Prospective Register of Systematic Reviews) under registration No. CRD42023466345.

### Literature search

Two reviewers independently conducted a literature search on the following online databases: PubMed (MEDLINE), Web of Science and Cochrane Library. The search query terms that were chosen to be used on PubMed were: (((laparoscopic esophagectomy) OR (minimally invasive esophagectomy) OR (video assisted esophagectomy) OR (thoracoscopic esophagectomy) OR ((esophagectomy[MeSH Terms]) AND ((laparoscopy[MeSH Terms]) OR (minimally invasive surgical procedures[MeSH Terms]) OR (surgery, video assisted[MeSH Terms]) OR (surgery, thoracoscopic[MeSH Terms]) OR (surgery, video assisted thoracoscopic[MeSH Terms])))) OR ((robotic esophagectomy) OR ((esophagectomy[MeSH Terms]) AND (robotics[MeSH Terms])))) AND (esophagus neoplasms[MeSH Terms]). Under the Web of Science database, the following query was used: [ALL = (laparoscopic esophagectomy) OR ALL = (minimally invasive esophagectomy) OR ALL = (video assisted esophagectomy) OR ALL = (thoracoscopic esophagectomy) OR ALL = (robotic esophagectomy)] AND [ALL = (esophagus cancer) OR ALL = (esophagus neoplasms)]. On Cochrane Library, the search entry was: laparoscopic esophagectomy in All Text OR minimally invasive esophagectomy in All Text OR thoracoscopic esophagectomy in All Text OR robotic esophagectomy in All Text AND esophagus neoplasms in All Text. Previous systematic reviews and meta-analysis were also consulted to identify additional studies of interest.

### Eligibility criteria

After the search results were exported, duplicated studies were detected and excluded. The resulting articles were assessed based on their titles and abstracts. Observational clinical studies and randomized controlled trials were deemed as relevant for inclusion when perioperative and short-term outcomes of robot-assisted and conventional minimally invasive curative-intent esophagectomies for resectable esophageal cancer patients were compared.

When available, full text articles were obtained and reviewed independently by two authors. Any discrepancies in selection were resolved by consensus. Studies were excluded according to the following criteria: (1) articles whose surgical technique did not include a robot-assisted thoracic phase esophagectomy; (2) comparison to a hybrid approach, instead of a conventional minimally invasive one; (3) studies that only assessed long-term outcomes; (4) absence of a comparison with a conventional minimally invasive technique. Indexed abstract posters and presentations, editorials, comments, and letters were also excluded.

### Data extraction

Eligible studies were assessed for data extraction by two independent reviewers. The following information was gathered from each study: author, publication year, country, study design and period, occurrence of propensity score matching (PSM), sample size, surgical procedure, histopathological tumor characteristics, neoadjuvant therapy prevalence, demographic data (age and sex) and short-term outcomes. Short-term outcomes were chosen according to previous systematic reviews: surgical outcomes—operating time, estimated blood loss, conversion to open procedure rate, harvested lymph nodes [total, mediastinal, abdominal, and along the left and right recurrent laryngeal nerve (RLN)] and 30- and 90-day mortality rates; and postoperative outcomes—anastomotic leakage, chyle leakage, recurrent laryngeal nerve palsy, pulmonary, cardiac and infectious complications and length of hospital stay (Tables 1, 2).

**Table 1:** Characteristics of studies included for analysis

No.	Author	Year	Country	Study design	Study period	PSM	Sample size		Surgical procedures
							RAMIE	cMIE	
1	Suda et al.	2012	Japan	OCS (P)	05.2009–08.2011	No	16	20	McKeown
2	Weksler et al.	2012	USA	OCS (R)	06.2008–10.2009	No	11	26	McKeown/Ivor-Lewis
3	Park et al.	2016	South Korea	OCS (R)	01.2006–06.2014	No	62	43	McKeown/Ivor-Lewis
4	Chao et al.	2018	China	OCS (R)	01.2013–05.2016	Yes	37	104	McKeown
5	Deng et al.	2018	China	OCS (P)	04.2016–01.2018	Yes	79	72	McKeown
6	He et al.	2018	China	OCS (R)	03.2016–12.2017	Yes	27	88	McKeown
7	Chen et al.	2019	China	OCS (R)	01.2016–01.2018	Yes	68	74	McKeown
8	Grimminger et al.	2019	Germany	OCS (P)	07.2015–08.2017	No	25	25	Ivor-Lewis
9	Motoyama et al.	2019	Japan	OCS (R)	12.2014–10.2018	No	21	38	NA
10	Zhang et al.	2019	China	OCS (R)	12.2014–06.2018	Yes	76	108	Ivor-Lewis
11	Chao et al.	2020	China	OCS (R)	06.2012–06.2017	No	39	67	McKeown
12	Gong et al.	2020	China	OCS (R)	01.2016–12.2018	No	91	144	McKeown
13	Meredith et al.	2020	USA	OCS (P)	1996–2016	No	144	95	Ivor-Lewis
14	Shirakawa et al.	2020	Japan	OCS (R)	11.2017–04.2019	Yes	66	90	NA
15	Tagkalos et al.	2020	Germany	OCS (P)	04.2016–04.2018	Yes	50	50	Ivor-Lewis
16	Yang et al.	2020	China	OCS (R)	11.2015–06.2018	Yes	280	372	McKeown
17	Ali et al.	2021	USA	OCS (R)	2010–2016	No	1543	5118	NA
18	Duan et al.	2021	China	OCS (R)	06.2017–12.2019	No	109	75	McKeown
19	Ninomiya et al.	2021	Japan	OCS (R)	04.2014–08.2020	Yes	30	30	NA
20	Oshikiri et al	2021	Japan	OCS (P)	2010–2020	Yes	51	353	McKeown
21	Tsunoda et al	2021	Japan	OCS (R)	01.2015–04.2020	Yes	49	85	McKeown/Ivor-Lewis
22	Balasubramanian et al	2022	India	OCS (R)	01.2015–09.2018	Yes	25	90	McKeown/Ivor-Lewis
23	Dezube et al.	2022	USA	OCS (R)	05.2016–08.2020	No	70	277	McKeown/Ivor-Lewis
24	Fujita et al.	2022	Japan	OCS (R)	01.2020–06.2021	Yes	55	134	NA
25	Kulkarni et al.	2022	India	OCS (R)	01.2016–12.2018	Yes	25	49	McKeown
26	Morimoto et al.	2022	Japan	OCS (R)	04.2018–03.2020	No	22	65	McKeown
27	Trung et al.	2022	Vietnam	OCS (R)	08.2018–04.2021	Yes	31	126	McKeown
28	van der Sluis et al.	2022	Germany	OCS (R)	01.2008–08.2019	No	123	91	McKeown/Ivor-Lewis
29	Yang et al.	2022	China	RCT	08.2017–12.2019	No	181	177	McKeown
30	Chouliaras et al.	2023	USA	OCS (R)	07.2013–11.2020	Yes	67	72	Ivor-Lewis
31	Jiang et al.	2023	China	OCS (R)	01.2016–01.2021	No	80	171	McKeown
32	Khaitan et al.	2023	USA	OCS (R)	2015–2019	Yes	1320	3524	McKeown/Ivor-Lewis
33	Narendra et al.	2023	Australia	OCS (R)	2005–2022	No	53	50	McKeown/Ivor-Lewis
34	Sun et al.	2023	China	OCS (R)	12.2020–11.2021	Yes	45	153	McKeown
35	Turner et al.	2023	USA	OCS (R)	2016–2020	No	234	926	McKeown/Ivor-Lewis

No.	Author	Year	Tumor histology									Neoadjuvant therapy				MINORS
			RAMIE				cMIE									
			SCC, n	SCC, %	AC, n	AC, %	SCC, n	SCC, %	AC, n		AC, %	RAMIE, n	RAMIE, %	MIE, n	MIE, %	
1	Suda et al.	2012	16	100.00	0	0.00	20	100.00	0		0.00	6	37.50	17	85.00	22
2	Weksler et al.	2012	0	0.00	10	90.91	3	11.54	23		88.46	4	36.36	10	38.46	21
3	Park et al.	2016	62	100.00	0	0.00	43	100.00	0		0.00	8	12.90	4	9.30	21
4	Chao et al.	2018	37	100.00	0	0.00	104	100.00	0		0.00	17	45.95	52	50.00	21
5	Deng et al.	2018	79	100.00	0	0.00	72	100.00	0		0.00	0	0.00	0	0.00	22
6	He et al.	2018	23	85.19	NA		80	90.91	NA			0	0.00	0	0.00	21
7	Chen et al.	2019	68	100.00	0	0.00	74	100.00	0		0.00	28	41.18	17	22.97	21
8	Grimminger et al.	2019	7	28.00	18	72.00	9	36.00	16		64.00	10	40.00	9	36.00	21
9	Motoyama et al.	2019	21	100.00	0	0.00	38	100.00	0		0.00	12	57.14	19	50.00	21
10	Zhang et al.	2019	74	97.37	0	0.00	107	99.07	0		0.00	0	0.00	0	0.00	21
11	Chao et al.	2020	38	97.44	1	2.56	65	97.01	2		2.99	39	100.00	67	100.00	21
12	Gong et al.	2020	86	94.51	NA		134	93.06		NA		20	21.98	28	19.44	21
13	Meredith et al.	2020	NA		NA		NA		NA			112	77.78	73	76.84	21
14	Shirakawa et al.	2020	NA		NA		NA		NA			30	45.45	51	56.67	20
15	Tagkalos et al.	2020	NA		NA		NA		NA			27	54.00	22	44.00	22
16	Yang et al.	2020	280	100.00	0	0.00	372	100.00	0		0.00	30	10.71	50	13.44	21
17	Ali et al.	2021	NA		NA		NA		NA			1147	74.34	3462	67.64	20
18	Duan et al.	2021	109	100.00	0	0.00	75	100.00	0		0.00	12	11.01	10	13.33	21
19	Ninomiya et al.	2021	NA		NA		NA		NA			20	66.67	25	83.33	19
20	Oshikiri et al	2021	45	88.24	6	11.76	325	92.07	28		7.93	30	58.82	246	69.69	22
21	Tsunoda et al	2021	46	93.88	2	4.08	81	95.29	4		4.71	29	59.18	57	67.06	21
22	Balasubramanian et al	2022	19	76.00	6	24.00	58	64.44	32		35.56	18	72.00	51	56.67	21
23	Dezube et al.	2022	NA		NA		NA		NA			NA		NA		21
24	Fujita et al.	2022	NA		NA		NA		NA			38	69.09	95	70.90	21
25	Kulkarni et al.	2022	15	60.00	10	40.00	40	81.63	9		18.37	17	68.00	43	87.76	21
26	Morimoto et al.	2022	21	95.45	1	4.55	63	96.92	2		3.08	10	45.55	36	55.39	21
27	Trung et al.	2022	31	100.00	0	0.00	126	100.00	0		0.00	NA		NA		19
28	van der Sluis et al.	2022	20	16.26	85	69.11	24	26.37	51		56.04	105	85.37	60	65.93	20
29	Yang et al.	2022	181	100.00	0	0.00	177	100.00	0		0.00	39	21.55	37	20.90	NA
30	Chouliaras et al.	2023	7	10.45	60	89.55	5	6.94	67		93.06	54	80.60	64	88.89	21
31	Jiang et al.	2023	80	100.00	0	0.00	171	100.00	0		0.00	80	100.00	171	100.00	21
32	Khaitan et al.	2023	NA		NA		NA		NA			NA		NA		21
33	Narendra et al.	2023	NA		NA		NA		NA			40	75.47	142	59.17	20
34	Sun et al.	2023	41	91.11	1	2.22	139	90.85	7		4.58	21	46.67	85	55.56	21
35	Turner et al.	2023	NA		NA		NA		NA			163	69.67	640	69.11	21

**Table 2:** Patients' demographics

No.	Author	Year	Age (RAMIE)		Age (MIE)		Sex (RAMIE)			Sex (MIE)		
			Mean	SD	Mean	SD	M	F	Pt	M	F	Pt
1	Suda et al.	2012	67.25	9.33	64.5	7.76	15	1	16	15	5	20
2	Weksler et al.	2012	58.7	8.5	64.3	11.3	8	3	11	20	6	26
3	Park et al.	2016	64.3	8	66.2	7.4	57	5	62	40	3	43
4	Chao et al.	2018	58.6	10.13	54.1	7.71	34	3	37	97	7	104
5	Deng et al.	2018	61.6	7	61.2	8.9	64	15	79	54	18	72
6	He et al.	2018	61	8	62.9	8.3	20	7	27	61	27	88
7	Chen et al.	2019	61.9	8.5	61.3	8.2	53	15	68	59	15	74
8	Grimminger et al.	2019	61.1	11.1	63	8.7	22	3	25	19	6	25
9	Motoyama et al.	2019	61.5	8.47	64	6.09	19	2	21	32	6	38
10	Zhang et al.	2019	61.8	7.7	61.3	7.7	59	17	76	85	23	108
11	Chao et al.	2020	57.41	8.59	54.55	7.93	35	4	39	65	2	67
12	Gong et al.	2020	60.04	NA	60.22	NA	78	13	91	130	14	144
13	Meredith et al.	2020	66	10	62	9	113	31	144	81	14	95
14	Shirakawa et al.	2020	66.67	10.61	67	9.04	56	10	66	74	16	90
15	Tagkalos et al.	2020	62	NA	64	NA	NA			NA		
16	Yang et al.	2020	63.1	7.3	63.9	7.8	230	50	280	302	70	372
17	Ali et al.	2021	63.61	9.47	63.74	9.32	1287	256	1543	4235	883	5118
18	Duan et al.	2021	60	6.1	61.1	6.6	90	19	109	65	10	75
19	Ninomiya et al.	2021	65	7.84	63	6.87	22	8	30	23	7	30
20	Oshikiri et al.	2021	64.75	7.77	60.75	9.43	34	17	51	301	52	353
21	Tsunoda et al.	2021	64.25	9.62	64	8.6	43	6	49	63	22	85
22	Balasubramanian et al.	2022	61.88	9.83	64.51	12.21	17	8	25	52	38	90
23	Dezube et al.	2022	66.5	8.01	60.5	10.56	57	13	70	226	51	277
24	Fujita et al.	2022	68.9	10.1	68.8	7.4	42	13	55	111	23	134
25	Kulkarni et al.	2022	59.2	8.3	56.1	11.1	13	12	25	26	23	49
26	Morimoto et al.	2022	67	6	67	8	19	3	22	53	12	65
27	Trung et al.	2022	59	6.4	57.9	7.7	31	0	31	123	3	126
28	van der Sluis et al.	2022	59.5	11.67	65	8.11	107	16	123	67	24	91
29	Yang et al.	2022	62	5.92	60.75	6.12	156	25	181	150	27	177
30	Chouliaras et al.	2023	60.3	10.28	64.9	8.65	57	10	67	64	8	72
31	Jiang et al.	2023	63.67	6.79	62.33	7.48	68	12	80	146	25	171
32	Khaitan et al.	2023	NA		NA		1086	234	1320	2820	704	3524
33	Narendra et al.	2023	NA		NA		44	9	53	194	46	240
34	Sun et al.	2023	64.25	2.72	64	2.08	30	15	45	118	35	153
35	Turner et al.	2023	64.67	9.7	64.67	9.65	198	36	234	759	167	926

### Quality assessment

The risk of bias and study quality assessment was performed using the MINORS (Methodological Index for Non-Randomized Studies) scale [25] in the observational clinical studies eligible for analysis. As for randomized controlled trials, the Cochrane Risk of Bias 2 tool (RoB2) [26] was used. It was conducted by two authors, and any disagreements were solved through discussion. MINORS scoring system comprises 12 items, 8 methodological and 4 additional criteria applied to comparative studies. In regards to methodology, studies were scored 0 (not reported), 1 (reported but inadequate) or 2 (reported and adequate) points, according to the description of a clearly stated aim, inclusion of consecutive patients, involvement of a prospective collection of data, existence of endpoints appropriate to the aim of the study, performance of an unbiased assessment of the study endpoint, admittance of a follow-up period appropriate to the aim of the study, registration of a loss to follow-up below 5% and a prospective calculation of the study size. The 4 additional criteria applied to comparative studies used the same scoring system, assessing the existence of an adequate and contemporary control group, without confounding factors, and the presence of an adequate statistical analysis (Table 1 and Supplementary Table 1).

### Statistical analysis

The meta-analysis was conducted using Review Manager (Version 5.4.1).

Results related to continuous outcomes were presented as mean differences (MD) with 95% confidence intervals (CI), by using the generic inverse variance method. Regarding dichotomous outcomes, results were presented as risk ratios (RR) with 95% CI, also using the generic inverse variance method. There were studies that reported their outcomes as median and range. In these cases, mean and standard deviation were estimated using a method described by Wan et al. [27]. Statistical significance was assessed using an alpha (*α*) level of 0.05. Heterogeneity was explored using the *I*^2^ measure and Cochran’s *Q* test, using a significance cutoff point of 0.10. We applied a random effects model due to the clinical heterogeneity of the included studies. PSM data were also analyzed to eliminate possible causes of heterogeneity. Publication bias among included studies was investigated by using funnel plots, provided as Supplementary File 1.

## Results

### Study selection

**Fig. 1:**
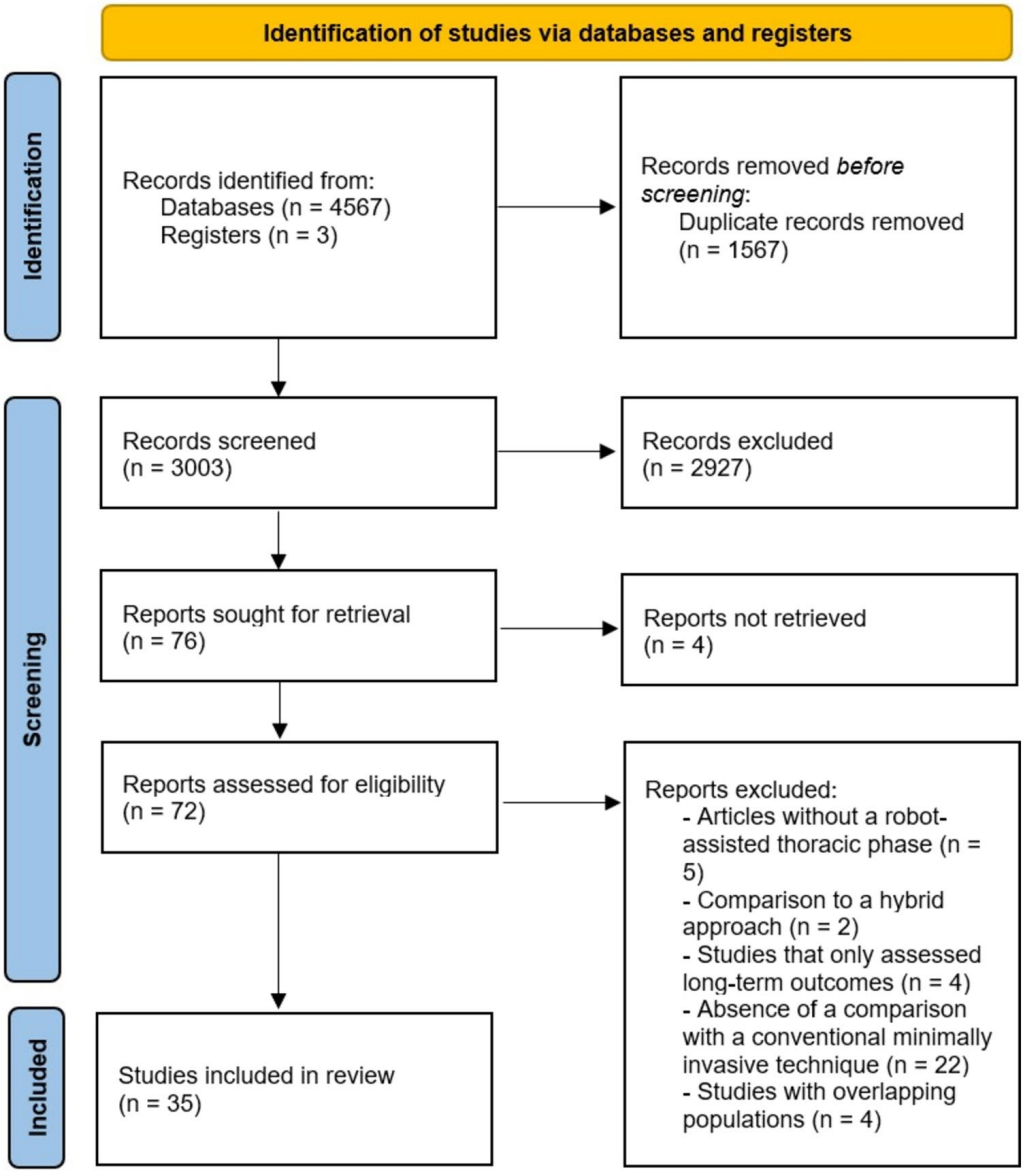
PRISMA flow diagram

The conducted literature search of PubMed, Web of Science and Cochrane Library resulted in 4567 articles selected for review. Duplicated studies were excluded, resulting in 3003 records whose titles and abstracts were screened. Upon review, 72 full-text articles were assessed for eligibility. From these, 37 studies were excluded according to inclusion and exclusion criteria. Previous systematic reviews yielded 3 additional studies after manual screening of relevant references. Therefore, 35 full-text studies were included for quality assessment and analysis (Fig. 1). Demographic data and details from the included studies are presented in Table 1. OCS were more prevalent, with 34 results [28–61], and 1 RCT [62] was also included. A total of 18,187 participants were analyzed, of which 5205 underwent RAMIE, and 12,982 were operated under cMIE.

### Quality assessment

All included studies revealed a fair methodological quality. The median score in the MINORS scale for OCS was 21, ranging from 19 to 22 points. As for the included RCT, the RoB2 tool calculated a low risk of bias judgement (Table 1 and Supplementary Table 1).

### Surgical outcomes

#### Operating time

**Fig. 2:**
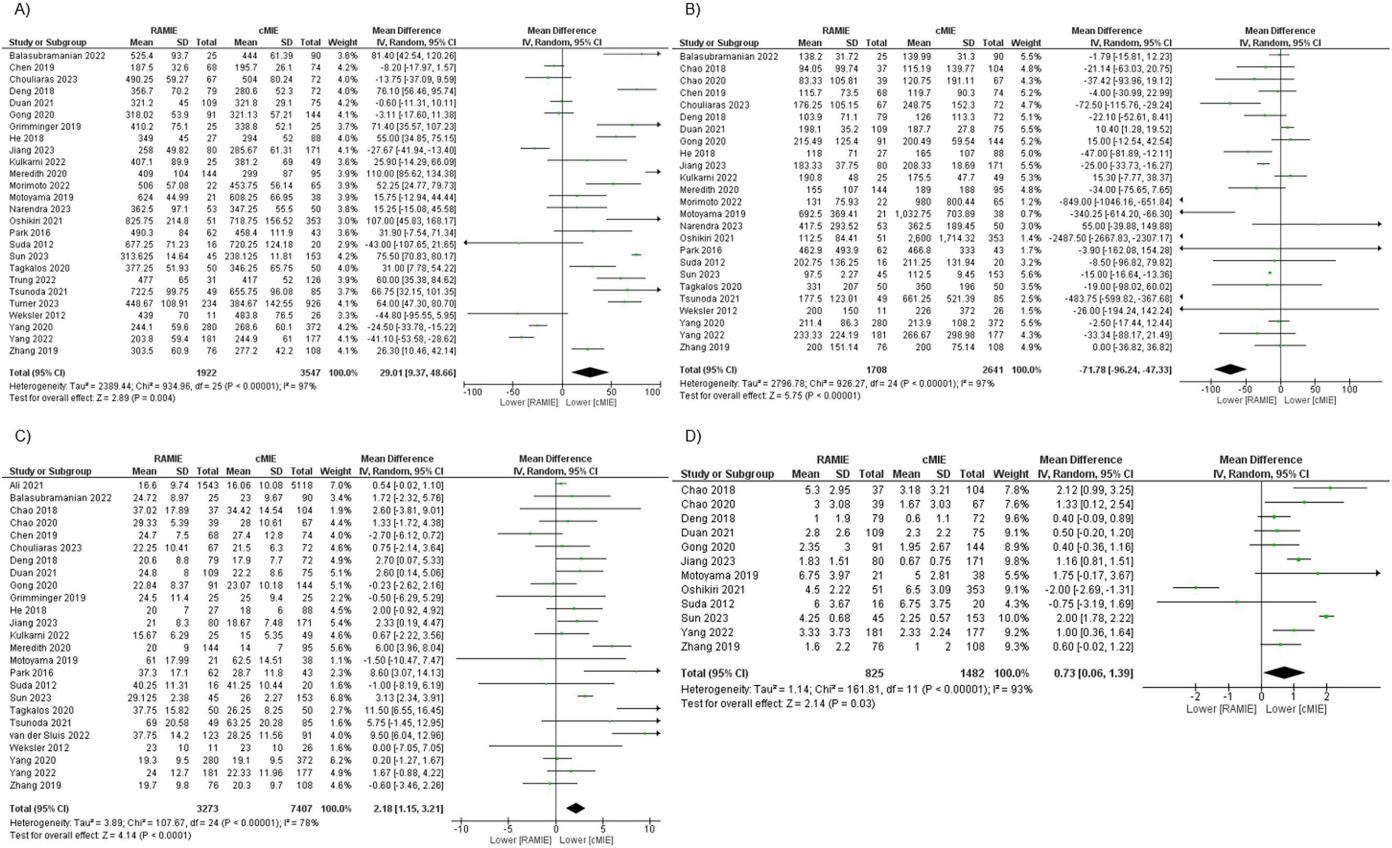
Meta-analysis of surgical outcomes: **a** operating time; **b** estimated blood loss; **c** harvested lymph nodes, TOTAL; **d** harvested lymph nodes, left RLN

The conducted meta-analysis gathered 26 studies which reported each operating time. The test for overall effect concluded that cMIE has a significantly shorter operating time when compared to RAMIE (MD 29.01, *p* = 0.004 [95% CI 9.37, 48.66], *I*^2^ = 97%, *p* < 0.00001). Mean operating time was 395.72 min in the cMIE group and 424.91 min in the RAMIE group (Fig. 2).

#### Estimated blood loss

Estimated blood loss was assessed and measured in 25 studies. It has appeared to be significantly less in RAMIE when compared to cMIE (MD − 71.78, *p* < 0.00001 [95% CI − 96.24, − 47.33], *I*^2^ = 97%, *p* < 0.00001). Mean estimated blood loss was 209.68 mL in the RAMIE group and 387.18 mL in the cMIE group (Fig. 2).

#### Conversion to open procedure rate

This outcome was included in 18 studies and did not show any statistically significant difference between the two groups (RR 0.64, *p* = 0.14 [95% CI 0.36, 1.16], *I*^2^ = 80%, *p* < 0.00001). Conversion to open procedure had a rate of 4.87% (197/4047) in the RAMIE group and of 8.22% (857/10431) in the cMIE group.

#### Harvested lymph nodes, TOTAL

The total number of harvested lymph nodes during the procedures was recorded and presented in 25 studies. Mean total number of harvested lymph nodes in the RAMIE group was 28.89 and 26.61 in the cMIE group. Their difference was statistically significant, favoring RAMIE (MD 2.18, *p* < 0.0001 [95% CI 1.15, 3.21], *I*^2^ = 78%, *p* < 0.00001) (Fig. 2).

#### Harvested lymph nodes, MEDIASTINAL

The number of mediastinal harvested lymph nodes was measured in 13 studies. There was no statistically significant difference between the two groups (MD − 0.76, *p* = 0.46 [95% CI − 2.77, 1.25], *I*^2^ = 91%, *p* < 0.00001). Mean number of mediastinal harvested lymph nodes was 18.72 in the RAMIE group and 19.61 in the cMIE group.

#### Harvested lymph nodes, ABDOMINAL

This outcome was reported in 10 studies. The mean number of abdominal harvested lymph nodes was 9.38 in the RAMIE group and 8.85 in the cMIE group, showing no statistically significant difference between these groups (MD 0.13, *p* = 0.66 [95% CI − 0.47, 0.73], *I*^2^ = 58%, *p* = 0.01).

#### Harvested lymph nodes, LEFT RLN

Harvested lymph nodes along the left RLN were assessed in 12 studies. The mean yield of these nodes was 3.56 in the RAMIE group and 2.85 in the cMIE group, favoring RAMIE (MD 0.73, *p* = 0.03 [95% CI 0.06, 1.39], *I*^2^ = 93%, *p* < 0.00001) (Fig. 2).

#### Harvested lymph nodes, RIGHT RLN

As for the harvested lymph nodes along the right RLN, these were included in 9 articles, without evidence of a statistically significant difference between the two groups (MD 0.12, *p* = 0.53 [95% CI − 0.26, 0.51], *I*^2^ = 86%, *p* < 0.00001). Mean yield of these nodes was of 2.23 in the RAMIE group and of 2.12 in the cMIE group.

#### 30-day mortality rate

Twenty studies reported their 30-day mortality rate for each procedure, being 1.63% (44/2707) in the RAMIE group and 1.87% (117/6244) in the cMIE group. There was no statistically significant difference between the two groups (RR 1.03, *p* = 0.88 [95% CI 0.73, 1.44], *I*^2^ = 0%, *p* = 0.53).

#### 90-Day Mortality rate

This outcome was measured in 18 studies. The rate of 90-day mortality was 3.55% (106/2987) in the RAMIE group and 4.84% (336/6946) in the cMIE group, showing no statistically significant difference between the two groups (RR 0.95, *p* = 0.66 [95% CI 0.77, 1.18], *I*^2^ = 0%, *p* = 0.93).

### Postoperative outcomes

#### Anastomotic leakage

**Fig. 3:**
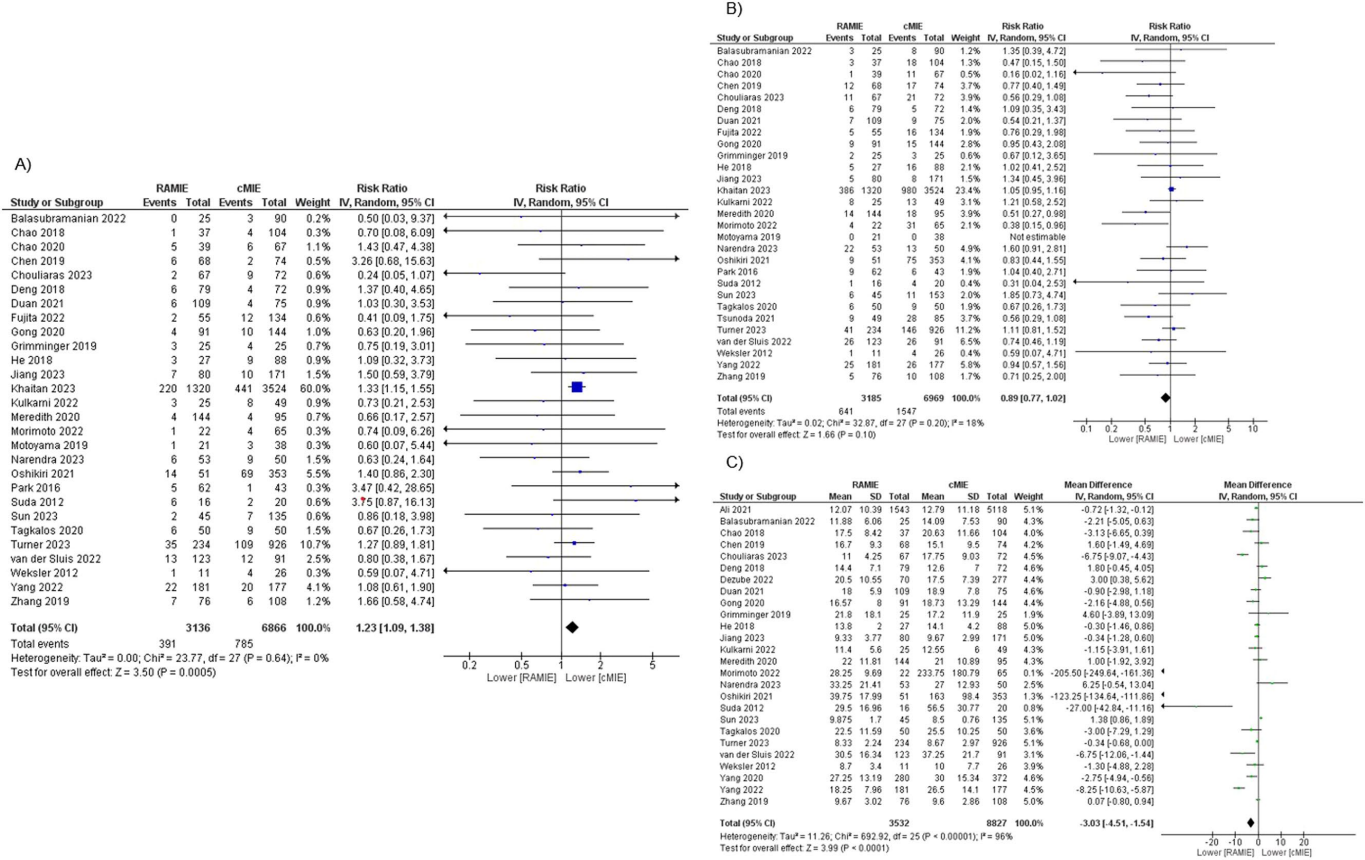
Meta-analysis of postoperative outcomes: **a** anastomotic leakage; **b** pulmonary complications; **c** length of hospital stay

Anastomotic leakage was measured and recorded in 28 studies. The rate of this complication was 12.47% (391/3136) in the RAMIE group and 11.43% (785/6866) in the cMIE group, favoring cMIE (RR 1.23, *p* = 0.0005 [95% CI 1.09, 1.38], *I*^2^ = 0%, *p* = 0.64) (Fig. 3).

#### Chyle leakage

Nineteen studies reported this outcome. There was no statistically significant difference between the two groups (RR 1.07, *p* = 0.74 [95% CI 0.72, 1.60], *I*^2^ = 13%, *p* = 0.30). The rate of chyle leakage was 2.82% (69/2443) in the RAMIE group and 3.84% (197/5135) in the cMIE group.

#### Recurrent laryngeal nerve palsy

RLN palsy was included in 23 studies. The rate of this event was 8.94% (237/2652) in the RAMIE group and 7.63% (423/5541) in the cMIE group, with no statistically significant difference between the two (RR 0.96, *p* = 0.62 [95% CI 0.82, 1.13], *I*^2^ = 7%, *p* = 0.36).

#### Pulmonary complications

Pulmonary complications were assessed in 29 studies. The rate of this complication was 20.13% (641/3185) in the RAMIE group and 22.20% (1547/6969) in the cMIE group. There was no statistically significant difference between the two groups (RR 0.89, *p* = 0.10 [95% CI 0.77, 1.02], *I*^2^ = 18%, *p* = 0.20) (Fig. 3).

#### Cardiac complications

Cardiac complications were included in 20 studies. The rate of this complication was 14.02% (365/2604) in the RAMIE group and 15.74% (823/5228) in the cMIE group. There was no statistically significant difference between the two groups (RR 1.01, *p* = 0.88 [95% CI 0.86, 1.19], *I*^2^ = 3%, *p* = 0.42).

#### Infectious complications

Seventeen studies reported infectious complications. The rate of this event was 2.53% (31/1223) in the RAMIE group and 3.18% (50/1572) in the cMIE group. These results showed no statistically significant difference between them (RR 0.97, *p* = 0.92 [95% CI 0.61, 1.56], *I*^2^ = 0%, *p* = 0.52).

#### Length of hospital stay

This outcome was included in 26 studies. The mean length of hospital stay was of 18.57 days in the RAMIE group and 33.11 days in the cMIE group. Meta-analysis favors RAMIE (MD − 3.03, *p* < 0.0001 [95% CI − 4.51, − 1.54], *I*^2^ = 96%, *p* < 0.00001) (Fig. 3).

### Propensity Score Matching Analysis

Propensity Score Matching Analysis information is gathered under Table 3. It was not possible to conduct a meta-analysis for the 30-Day Mortality rate outcome, as only one event was recorded in the cMIE group, in one of the six included studies. RAMIE showed superiority over cMIE in estimated blood loss, total number of harvested lymph nodes along the left RLN, and over pulmonary complications. Regarding operating time, cMIE was able to be executed under a lower amount of time. The rest of the measured outcomes presented no statistically significant differences between the two groups.

**Table 3:** Propensity score matching results

Outcomes	No. of studies	No. of patients		Overall effect size (MD/RR)	95% CI of overall effect	*p*	Heterogeneity	
		RAMIE	cMIE				*I*^2^ (%)	*p*
Surgical outcomes								
Operating time	13	776	805	41.37	13.95 to 68.78	0.003	96	<0.00001
Estimated blood loss	15	862	862	− 25.14	− 43.18 to − 7.11	0.006	81	<0.00001
Conversion to open procedure rate	10	637	637	0.44	0.02 to 9.30	0.6	68	0.08
Harvested lymph nodes, TOTAL	13	781	781	1.79	0.40 to 3.18	0.01	65	0.0005
Harvested lymph nodes, MEDIASTINAL	8	578	607	0.1	− 1.11 to 1.31	0.87	47	0.07
Harvested lymph nodes, ABDOMINAL	4	400	400	0.78	− 0.03 to 1.59	0.06	56	0.08
Harvested lymph nodes, LEFT RLN	5	312	312	0.95	0.04 to 1.86	0.04	90	<0.00001
Harvested lymph nodes, RIGHT RLN	4	195	195	0.15	− 0.15 to 0.46	0.33	18	0.3
30-day mortality rate	6	282	282					
90-day mortality rate	8	537	537	0.68	0.25 to 1.86	0.45	0	0.9
Postoperative outcomes								
Anastomotic leakage	14	837	837	0.89	0.67 to 1.19	0.44	0	0.78
Chyle leakage	9	598	598	0.88	0.42 to 1.86	0.74	0	0.95
Recurrent laryngeal nerve palsy	15	850	850	0.81	0.54 to 1.20	0.3	66	0.0002
Pulmonary complications	16	912	912	0.7	0.56 to 0.86	0.001	0	0.74
Cardiac complications	9	632	632	1.06	0.64 to 1.77	0.81	5	0.39
Infectious complications	8	591	591	1.03	0.46 to 2.24	0.93	0	0.87
Length of hospital stay	13	787	787	− 1.44	− 3.20 to 0.31	0.11	90	<0.00001

## Discussion

This systematic review and meta-analysis directly compares relevant short-term outcomes between robot-assisted and conventional minimally invasive esophagectomies in esophageal cancer patients. Previous meta-analysis [17–24] concluded that RAMIE is a superior surgical approach when compared to cMIE regarding blood loss, number of harvested lymph nodes, RLN palsy and pulmonary complications, while operating time is shorter in cMIE. Nonetheless, some of these results are also associated with a high percentage of heterogeneity among the studies, compromising their validity. With the publication of further data and literature, it became necessary to perform an updated meta-analysis to not only confirm the safety and feasibility of RAMIE, but also to establish new conclusions when compared to cMIE.

Our analysis concluded that both surgical approaches result in similar rates of conversion to open procedure, mean number of harvested lymph nodes in the mediastinum, abdomen and along the right RLN, 30- and 90-day mortality rates, chyle leakage, RLN palsy as well as cardiac and infectious complication rates.

A significantly shorter operating time and a lower rate of anastomotic leakage is seen in cMIE, while estimated blood loss, total number of harvested lymph nodes and along the left RLN, pulmonary complications and length of hospital stay are outcomes that favor RAMIE.

### Surgical outcomes

#### Operating time

The mean operating time revealed to be around 29 min shorter in the cMIE group, when compared to RAMIE. This result is coherent with three previous meta-analysis [17, 18, 21]. There are two main reasons that explain a longer duration of surgical procedures with RAMIE.

Firstly, port setup, docking and repositioning of instruments and uninstallation of devices all account for extra time in an inexperienced surgical team. Secondly, most of the reviewed studies reported their data with surgeons that had not yet completed their learning curve in this specific type of surgery. Recent studies suggest that this learning curve is achieved in surgeons after 20–22 cases of successful RAMIE, with a previously vast experience in cMIE, attaining proficiency, lessening complications, and reducing operating time [59, 60].

Upon examination of our forest plot, we conclude that three of the included studies demonstrate that the robotic surgery group operated in a significantly shorter timeframe [42, 57, 62]. This is due to a docking time of only around 5 min in each of these scenarios and a fully experienced surgical team, both in assistants that perform equally in RAMIE and cMIE and also surgeons that have completed their learning curve. Therefore, it has been proven that once surgeons attain their proficiency with the equipment, RAMIE offers advantages in the manipulation of instruments and camera control that enable increased efficiency in the operation [62].

Analysis of PSM data from studies that performed this procedure leads us to a similar conclusion. The mean difference was larger, achieving over 41 min, favoring cMIE. Again, one of these studies [42] had statistically significant shorter operating times favoring RAMIE, explained by the same reasons.

#### Estimated blood loss

Our analysis suggests that RAMIE is associated with a reduction in estimated blood lost during the surgical procedures. The difference of blood loss between the two approaches is approximately 72 mL, favoring RAMIE, whose clinical significance may be residual. Previous meta-analyses present the same conclusion [17–19]. This result accounts for a majority of included studies that show no statistically significant difference between the two groups, but with several outliers that describe major complications of episodical events in the cMIE group. Therefore, this conclusion is to be taken carefully, taking these factors into consideration and an elevated heterogeneity among studies.

Nonetheless, this difference may be explained due to RAMIE’s improved instrument dexterity navigating in the surgical field and its tremor filtering systems. Lymph node dissection is made easier, damaging less blood vessels along the procedure [19].

PSM data suggest an even smaller, but still significant difference. The mean difference between the two procedures is approximately 25 mL. Such a small amount of blood lost might not be clinically relevant to the patient’s morbidity perioperatively.

#### Conversion to open procedure rate

This outcome showed no statistically significant difference between RAMIE and cMIE. Three of the included studies had a higher rate of conversion to open procedure [40, 42, 43]. These were explained by all the advantages that the robotic system has to offer in attaining proficiency in the procedures. Aside from these studies, all others had comparable results, enhancing the need for further data on this topic.

Upon analysis of PSM information, the same conclusion is achieved. A wider population needs to be assessed in order to have more information regarding conversion.

#### Harvested lymph nodes, TOTAL, MEDIASTINAL, ABDOMINAL, LRLN, RRLN

Lymphadenectomy has become an essential element of the primary management of esophagectomies. Due to a narrow operative field, lymph node dissection is especially challenging along each of the RLNs, being the surgeon’s intent to harvest as many lymph nodes as possible, without damaging the RLN or adjacent structures. This procedure is important to both categorize the tumor according to the TNM classification, but also apply curative-intent treatment [19].

Harvested lymph nodes had statistically significant results in total numbers and in those that were yielded along the left RLN, both favoring the RAMIE group. Previous meta-analyses discuss similar findings [19, 23, 24]. Nonetheless, our results present high heterogeneity among studies, which leads to questions regarding the external validity of these findings.

The robotic group was favored in 8 of the 25 included studies when total lymph nodes dissected were recorded. The mean difference between the groups was of approximately two additional lymph nodes. Along the left RLN region, our search included 12 studies. Results also favored RAMIE, with five of these that individually favored the robotic approach. RAMIE yielded approximately one additional lymph node over cMIE. Lymph node count that were dissected in the mediastinum, abdomen, and along the right RLN did not show differences between the two groups. Improved anatomical dissection precision, featured in RAMIE, accounts for a larger number of lymph node yield in these regions. This is due to a magnified surgical field of view and improved imaging, with improved dexterity and flexibility of instruments and physiological tremor filtering systems. Regarding the left upper mediastinum, where the left RLN is dissected, the robotic approach is able to provide the surgeon with an upgraded vision and field of view, promoting the identification of the nerve. This, along with an improved posture and comfort when operating, enables the surgeon to have a greater ease of access to such a narrow and out of reach area like the left upper mediastinum.

A minimum of 15 lymph nodes necessary for accurate staging [63] was achieved in all studies, which reflects the quality of oncologic resection and staging. Whether these additional nodes using the RAMIE technique represent better long-term resection is still unclear. Clinically, a more relevant measure to be reported by authors would be the total number of positive lymph nodes harvested, as these directly influence the disease’s course of action and prognosis.

Patients that underwent neoadjuvant therapy before surgery often present with edema and fibrosis in the affected areas. In these patients, RAMIE performed particularly better than cMIE due to its advantageous features [62].

Studies that performed PSM showed similar results, further increasing evidence regarding RAMIE’s superiority in the dissection of lymph nodes during esophagectomies.

#### 30- and 90-day mortality rates

Early mortality rates had no statistically significant differences between the two surgical approaches. The same conclusion was attained by previous meta-analyses [17, 18, 20–24]. 30-day mortality rates are below 2% and 90-day rates are below 5% in both groups. These two indicators reflect greatly on the quality of surgery performed on esophageal cancer patients and the occurrence of major complications after the procedure [64].

### Postoperative outcomes

#### Anastomotic and chyle leakage

Anastomotic leakage is one of the most impactful complications that may occur after an esophagectomy, as it leads to high rates of morbidity and mortality in such patients. Several important factors are highlighted when analyzing predisposing conditions that lead to this complication. Preoperatively, diseases that compromise the perfusion of the anastomosis are congestive heart failure, coronary artery disease, diabetes, smoking, and hypertension. With regard specifically to the surgery, the anastomotic technique, the location of the anastomosis, the type of conduit and its location all contribute to the quality of the performed anastomosis and therefore influence its longevity [65].

In our study, cMIE had a statistically significant lower rate of anastomotic leakage. This is highly influenced by a single OCS that had a large study population from a national database [58]. One of their reported limitations is the lack of information regarding the anastomotic technique used in all procedures, which directly influences this outcome. Furthermore, this study did not exclude the surgical learning curve of minimally invasive operations, which is inherently accompanied by a higher rate of complications in such a critical phase of the surgery. If this study is removed from the analysis, results are no longer statistically significant, favoring no technique.

On the other hand, chyle leakage, another important factor in postoperative morbidity, did not favor any approach. Heterogeneity was low, and there was no selection bias upon funnel plot analysis.

Information from PSM studies corroborates these results, showing similar rates of anastomotic and chyle leakage.

#### Recurrent laryngeal nerve palsy

Thoracic surgeries, specifically esophagectomies, imply heavy esophageal manipulation and traction, which may damage RLN bilaterally. Neuropathy caused by stretching, compression, thermal and ischemic injury is a common complication that brings significant morbidity. Paralysis of the RLN leads to pulmonary complications, hoarseness, longer length of hospital stay, and long-term recovery, which in turn, deteriorate postoperative quality of life [66, 67].

Despite more extended lymph node dissection in RLNs in the robotic procedure, the occurrence of RLN palsy was similar in both groups. This result is also in accordance with previous meta-analyses [18–22, 24]. Therefore, one may argue that with RAMIE’s improved manipulation and visualization features, it is possible to attain better lymph node yields without compromising RLN damage and paralysis. It is also possible that once surgeons complete their learning curves in the technique, the rate of this complication decreases. There is also a need for further studies and information that differentiate the extent of nerve injury, for further characterization. PSM data lead us to the same conclusion.

#### Pulmonary, cardiac and infectious complications

Overall complications greatly reflect both the immediate response to such a major surgery, but also the prognosis and long-term development of the disease [68]. It has been proven that these short-term complications directly influence tumor progression and long-term survival. Minimally invasive surgical approaches and multidisciplinary case management are successful in the prevention of such outcomes [69].

In our unmatched pool of data, there was a great tendency towards lower pulmonary complication rates in the RAMIE approach, with a low level of heterogeneity among studies. This may be explained by the recent advantages that RAMIE has brought. Improved magnification and dexterity in the surgical field provide a better visualization and manipulation of ligaments and fasciae [70]. Due to this, the avoidance of dissection of the vagus nerve and preservation of increased segments of pulmonary parenchyma are better achieved. The vagus nerve is responsible for the regulation of essential pulmonary functions and mechanisms. The cough reflex, mucous production, bronchus diameter and regulation of inflammation and edema are all regulated by the vagus nerve [71]. Therefore, vagotomy will decrease the action of these response mechanisms to esophagectomy’s aggression and inflammatory response [72], leading to an increase in the rate of pulmonary complications. RAMIE may be able to overcome this unintended result and reduce the incidence of pneumonia, acute respiratory distress syndrome and other complications. Cardiac and infectious complications had similar rates of occurrence in the two surgical techniques. Recent meta-analyses present similar results [17, 18, 20, 22–24].

Analysis of PSM information further increase evidence of RAMIE’s superiority over cMIE in regards to pulmonary complications. There was a reduction of approximately 30% in the incidence of these complications. Conclusions regarding cardiac and infectious complications remained unaltered.

#### Length of hospital stay

Lower rates of postoperative complications directly influence a patient’s length of hospital stay after an esophagectomy [73]. As our results suggest a lower incidence of pulmonary complications in RAMIE patients, it is expected that those will require a shorter period of hospitalization.

Our analysis favors RAMIE to provide statistically significant shorter lengths of hospital stay, when compared to cMIE. Nonetheless, these results are to be taken with caution, as heterogeneity is high, and three studies [28, 46, 54] showed discrepant results when compared to the rest of the included ones. The calculated mean length of hospital stay was approximately 19 days in the RAMIE group and 33 days in the cMIE group. These are greatly influenced by Asian cultural and non-clinical factors that extend hospitalization further [74].

Upon analysis of PSM information, this difference is greatly attenuated and no longer statistically significant. The RAMIE group presented with a mean length of hospital stay of 17 days, and the cMIE group with 19 days. Some of the previous outlier cases are no longer taken into consideration and baseline patient characteristics are more similar. Nonetheless, there is still a high tendency towards a shorter length of hospital stay associated with the RAMIE procedure, which should be analyzed considering the context.

## Limitations

Our study acknowledges some limitations which may compromise the analysis of the attained results. First, all but one of the included studies are observational clinical studies, most of these being retrospective non-randomized comparative studies. Even though all studies present an assessed low risk of bias, patient selection bias is inherently present due to several cultural, clinical, and non-clinical factors of the preferred surgical approach. Furthermore, this systematic review includes studies that gather data from large national databases, from multiple institutions and different managing software. This results in a non-uniform way of coding and storing information, which may lead to unpredicted conclusions. Second, the wide majority of the reviewed studies represent the Asian population and local health policies and standards, which may not be generalizable globally. Third, heterogeneity was high in several measured outcomes, which may compromise the validity of the results. Varied reasons partially account for these differences, amongst which, surgical team’s and surgeons’ previous experience and position in the learning curve of the used surgical approach influence the most. Baseline demographics and clinical characteristics of patients in both groups also vastly impact heterogeneity. In fact, it was only possible to gather PSM information from 18 of the 35 included studies. Fourth, the analyzed studies included different surgical approaches to esophagectomy (McKeown and Ivor-Lewis), which may generate another degree of heterogeneity to the data.

## Conclusion

In conclusion, our study reinforces RAMIE’s safety and feasibility in the curative treatment of esophageal cancer patients. Analyzing the collected information, RAMIE shows superiority over cMIE in reducing blood lost during surgery, increasing the amount of dissected lymph nodes, decreasing pulmonary complications, and shortening periods of hospitalization after such an extensive and challenging surgery. Nonetheless, there seems to be a tendency towards shorter operative times and lower rates of anastomotic leakage using cMIE. Conversion to open surgery, 30- and 90-day mortality rates, chyle leakage, RLN palsy and cardiac and infectious complications did not show statistically significant differences between the two approaches.

Upon review of these results, RAMIE has indicated to be non-inferior to cMIE, with prospects of superiority in some fields. With the consolidation of the robotic approach, new opportunities will arise for the implementation of systems that enhance the surgical experience, such as haptic features, with kinesthetic and tactile feedback. Integration of preoperative imaging exams in the surgical field of view is also a possibility, overlapping anatomical structures with auxiliary images, which in turn facilitates the identification of dissection layers, tumor borders, and others. With the improvement of the surgeon’s comfort and confidence in decision-making, patients will be provided with a better standard of care.

Further studies, especially RCTs, are still necessary to overcome the limitations presented, in order to achieve definite conclusions regarding RAMIE’s current position in the treatment of esophageal cancer patients.

## Supplementary Information

Below is the link to the electronic supplementary material.

Supplementary file1 (DOCX 577 kb)

## Data Availability

No datasets were generated or analyzed during the current study.
