# Supplementary material for: Short-term outcomes of robot-assisted versus conventional minimally invasive esophagectomy for esophageal cancer: a systematic review and meta-analysis of 18,187 patients
Source: J Robot Surg. 2024 Mar 16;18(1):125. doi: 10.1007/s11701-024-01880-3 (PMC10944433; doi:10.1007/s11701-024-01880-3)
Supplement: Supplementary file 1 — Supplementary file1 (DOCX 577 kb) [file 11701_2024_1880_MOESM1_ESM.docx]

| No. | Author | Year | M1 | M2 | M3 | M4 | M5 | M6 | M7 | M8 | M9 | M10 | M11 | M12 | Total |
| --- | --- | --- | --- | --- | --- | --- | --- | --- | --- | --- | --- | --- | --- | --- | --- |
| 1 | Suda, K. et al | 2012 | 2 | 2 | 2 | 2 | 2 | 2 | 2 | 0 | 2 | 2 | 2 | 2 | 22 |
| 2 | Weksler, B. et al | 2012 | 2 | 2 | 1 | 2 | 2 | 2 | 2 | 0 | 2 | 2 | 2 | 2 | 21 |
| 3 | Park, S. et al | 2016 | 2 | 2 | 1 | 2 | 2 | 2 | 2 | 0 | 2 | 2 | 2 | 2 | 21 |
| 4 | Chao, Y.-K. et al | 2018 | 2 | 2 | 1 | 2 | 2 | 2 | 2 | 0 | 2 | 2 | 2 | 2 | 21 |
| 5 | Deng, H.-Y. et al | 2018 | 2 | 2 | 2 | 2 | 2 | 2 | 2 | 0 | 2 | 2 | 2 | 2 | 22 |
| 6 | He, H. et al | 2018 | 2 | 2 | 1 | 2 | 2 | 2 | 2 | 0 | 2 | 2 | 2 | 2 | 21 |
| 7 | Chen, J. et al | 2019 | 2 | 2 | 1 | 2 | 2 | 2 | 2 | 0 | 2 | 2 | 2 | 2 | 21 |
| 8 | Grimminger, P. et al | 2019 | 1 | 2 | 2 | 2 | 2 | 2 | 2 | 0 | 2 | 2 | 2 | 2 | 21 |
| 9 | Motoyama, S. et al | 2019 | 2 | 2 | 1 | 2 | 2 | 2 | 2 | 0 | 2 | 2 | 2 | 2 | 21 |
| 10 | Zhang, Y. et al | 2019 | 2 | 2 | 1 | 2 | 2 | 2 | 2 | 0 | 2 | 2 | 2 | 2 | 21 |
| 11 | Chao, Y.-K. et al | 2020 | 2 | 2 | 1 | 2 | 2 | 2 | 2 | 0 | 2 | 2 | 2 | 2 | 21 |
| 12 | Gong, L. et al | 2020 | 2 | 2 | 1 | 2 | 2 | 2 | 2 | 0 | 2 | 2 | 2 | 2 | 21 |
| 13 | Meredith, K. et al | 2020 | 2 | 2 | 2 | 2 | 2 | 2 | 2 | 0 | 2 | 2 | 1 | 2 | 21 |
| 14 | Shirakawa, Y. et al | 2020 | 2 | 2 | 1 | 2 | 2 | 2 | 2 | 0 | 2 | 1 | 2 | 2 | 20 |
| 15 | Tagkalos, E. et al | 2020 | 2 | 2 | 2 | 2 | 2 | 2 | 2 | 0 | 2 | 2 | 2 | 2 | 22 |
| 16 | Yang, Y. et al | 2020 | 2 | 2 | 1 | 2 | 2 | 2 | 2 | 0 | 2 | 2 | 2 | 2 | 21 |
| 17 | Ali, A. et al | 2021 | 2 | 2 | 1 | 2 | 2 | 2 | 2 | 0 | 2 | 2 | 1 | 2 | 20 |
| 18 | Duan, X. et al | 2021 | 2 | 2 | 1 | 2 | 2 | 2 | 2 | 0 | 2 | 2 | 2 | 2 | 21 |
| 19 | Ninomiya, I. et al | 2021 | 1 | 2 | 1 | 2 | 2 | 2 | 2 | 0 | 2 | 1 | 2 | 2 | 19 |
| 20 | Oshikiri, T. et al | 2021 | 2 | 2 | 2 | 2 | 2 | 2 | 2 | 0 | 2 | 2 | 2 | 2 | 22 |
| 21 | Tsunoda, S. et al | 2021 | 2 | 2 | 1 | 2 | 2 | 2 | 2 | 0 | 2 | 2 | 2 | 2 | 21 |
| 22 | Balasubramanian, S. et al | 2022 | 2 | 2 | 1 | 2 | 2 | 2 | 2 | 0 | 2 | 2 | 2 | 2 | 21 |
| 23 | Dezube, A. et al | 2022 | 2 | 2 | 1 | 2 | 2 | 2 | 2 | 0 | 2 | 2 | 2 | 2 | 21 |
| 24 | Fujita, T. et al | 2022 | 2 | 2 | 1 | 2 | 2 | 2 | 2 | 0 | 2 | 2 | 2 | 2 | 21 |
| 25 | Kulkarni, A. et al | 2022 | 2 | 2 | 1 | 2 | 2 | 2 | 2 | 0 | 2 | 2 | 2 | 2 | 21 |
| 26 | Morimoto, Y. et al | 2022 | 2 | 2 | 1 | 2 | 2 | 2 | 2 | 0 | 2 | 2 | 2 | 2 | 21 |
| 27 | Trung, L. et al | 2022 | 2 | 0 | 1 | 2 | 2 | 2 | 2 | 0 | 2 | 2 | 2 | 2 | 19 |
| 28 | van der Sluis, P. et al | 2022 | 2 | 2 | 1 | 2 | 2 | 2 | 2 | 0 | 2 | 1 | 2 | 2 | 20 |
| 29 | Yang, Y. et al | 2022 | NA | | | | | | | | | | | | |
| 30 | Chouliaras, K. et al | 2023 | 2 | 2 | 1 | 2 | 2 | 2 | 2 | 0 | 2 | 2 | 2 | 2 | 21 |
| 31 | Jiang, H. et al | 2023 | 2 | 2 | 1 | 2 | 2 | 2 | 2 | 0 | 2 | 2 | 2 | 2 | 21 |
| 32 | Khaitan, P. et al | 2023 | 2 | 2 | 1 | 2 | 2 | 2 | 2 | 0 | 2 | 2 | 2 | 2 | 21 |
| 33 | Narendra, A. et al | 2023 | 2 | 2 | 1 | 2 | 2 | 2 | 2 | 0 | 2 | 1 | 2 | 2 | 20 |
| 34 | Sun, H. et al | 2023 | 2 | 2 | 1 | 2 | 2 | 2 | 2 | 0 | 2 | 2 | 2 | 2 | 21 |
| 35 | Turner, K. et al | 2023 | 2 | 2 | 1 | 2 | 2 | 2 | 2 | 0 | 2 | 2 | 2 | 2 | 21 |

Supplementary Table 1 MINORS Quality Assessment


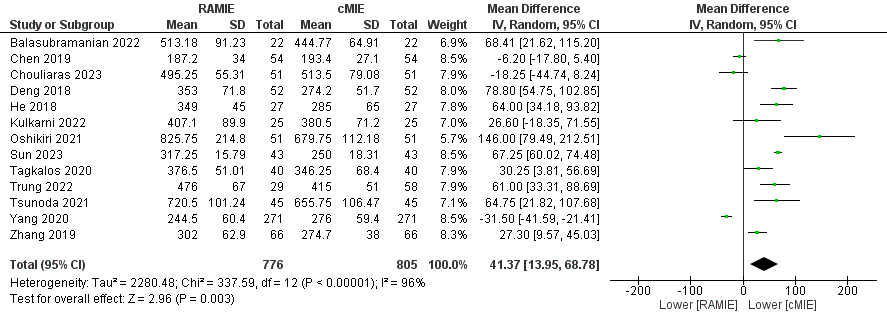


Supplementary Figure 1 Operating Time, PSM


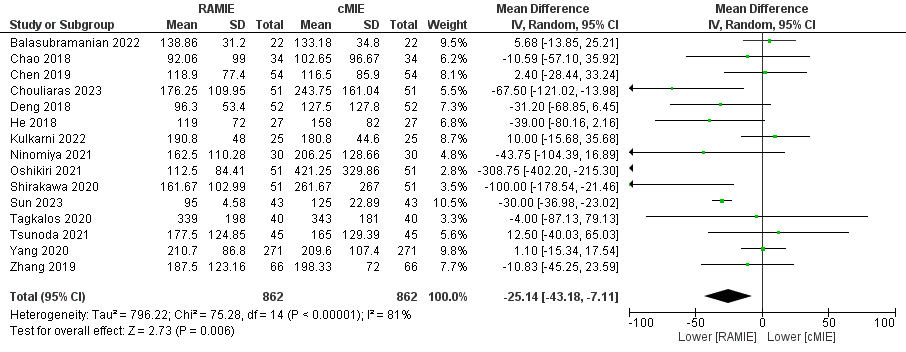


Supplementary Figure 2 Estimated Blood Loss, PSM


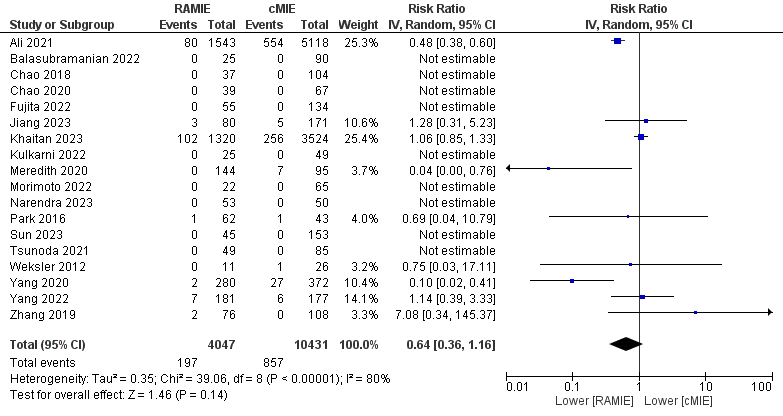


Supplementary Figure 3 Conversion to Open Procedure


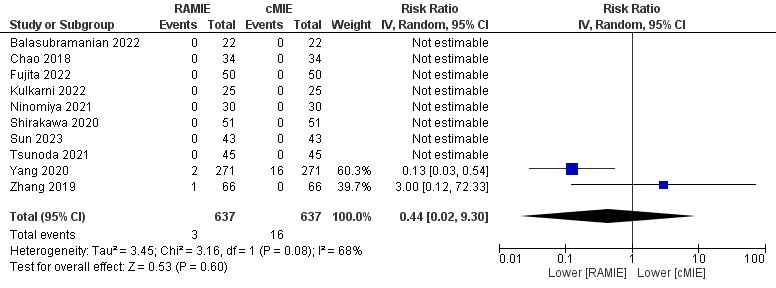


Supplementary Figure 4 Conversion to Open Procedure, PSM


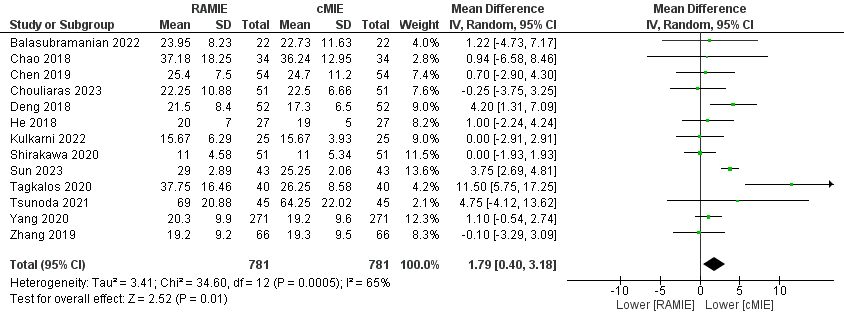


Supplementary Figure 5 Harvested Lymph Nodes, TOTAL, PSM


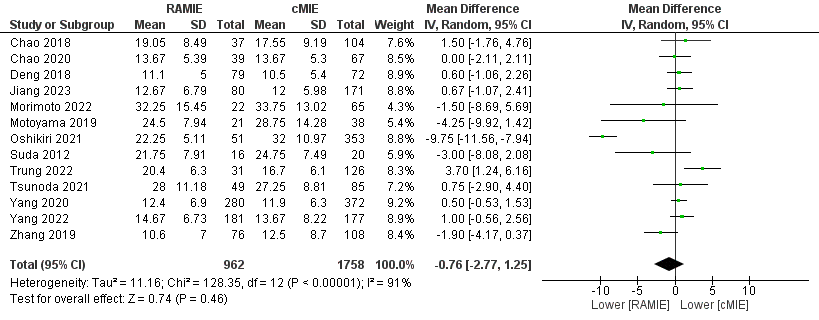


Supplementary Figure 6 Harvested Lymph Nodes, MEDIASTINAL


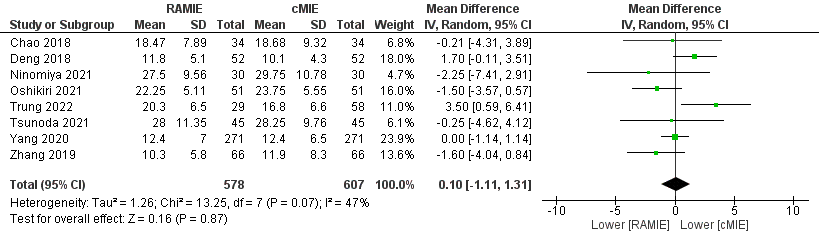


Supplementary Figure 7 Harvested Lymph Nodes, MEDIASTINAL, PSM


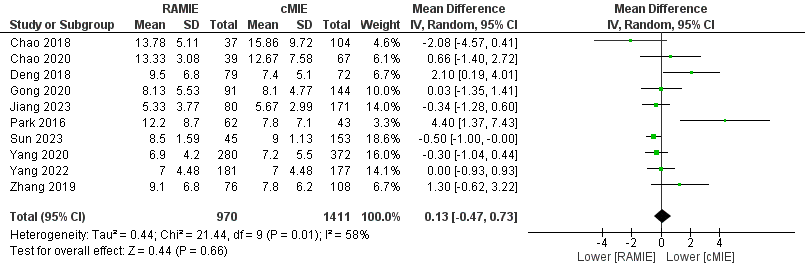


Supplementary Figure 8 Harvested Lymph Nodes, ABDOMINAL


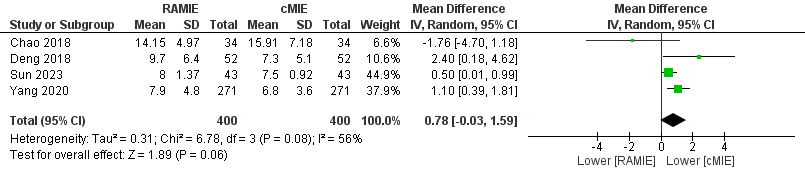


Supplementary Figure 9 Harvested Lymph Nodes, ABDOMINAL, PSM


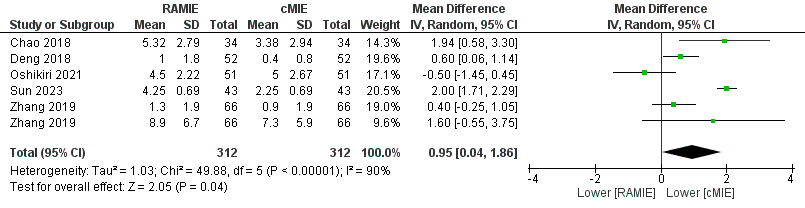


Supplementary Figure 10 Harvested Lymph Nodes, Left RLN, PSM


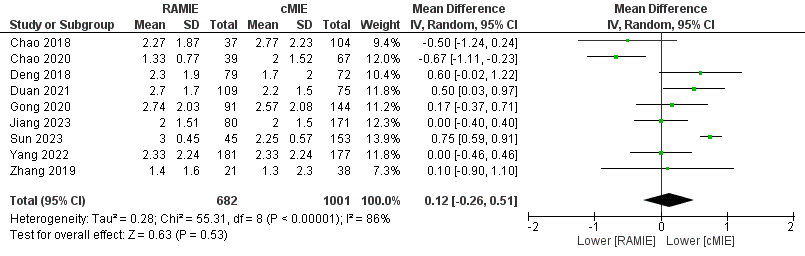


Supplementary Figure 11 Harvested Lymph Nodes, Right RLN


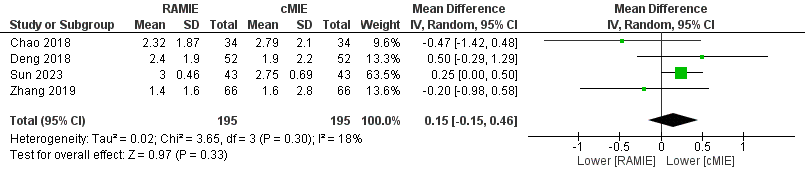


Supplementary Figure 12 Harvested Lymph Nodes, Right RLN, PSM


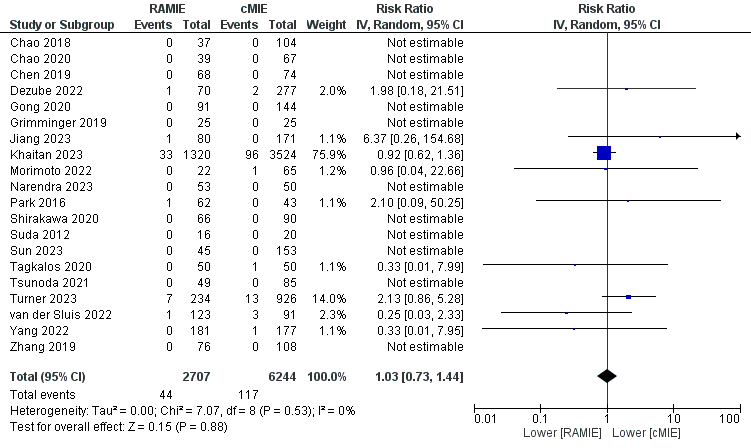


Supplementary Figure 13 30-Day Mortality


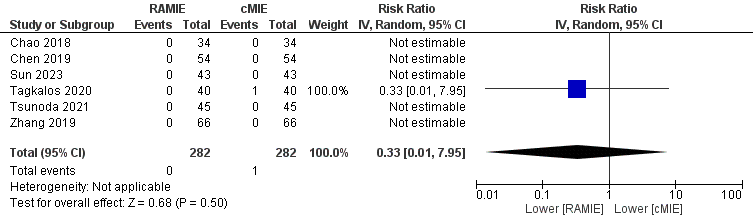


Supplementary Figure 14 30-Day Mortality, PSM


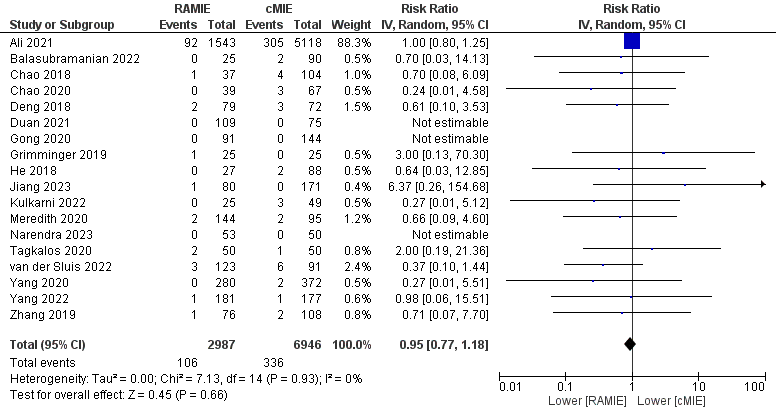


Supplementary Figure 15 90-Day Mortality


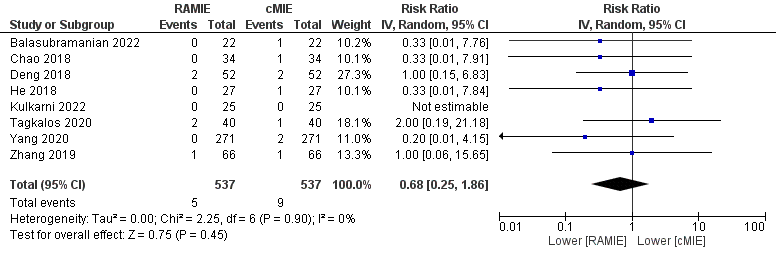


Supplementary Figure 16 90-Day Mortality, PSM


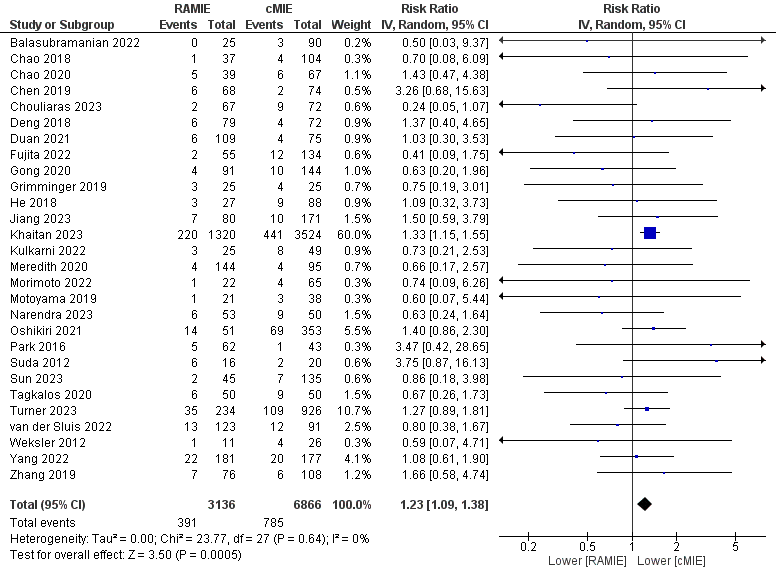


Supplementary Figure 17 Anastomotic Leakage


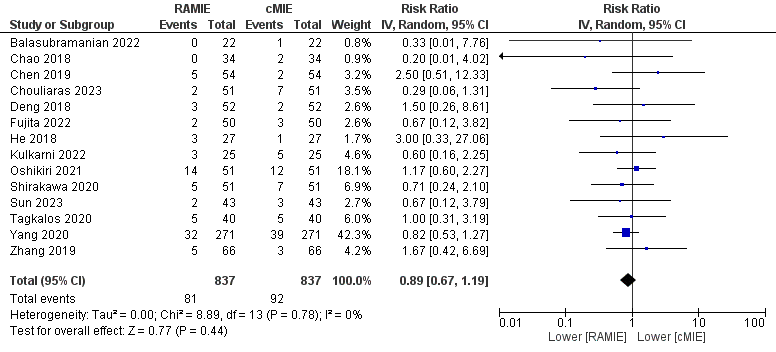


Supplementary Figure 18 Anastomotic Leakage, PSM


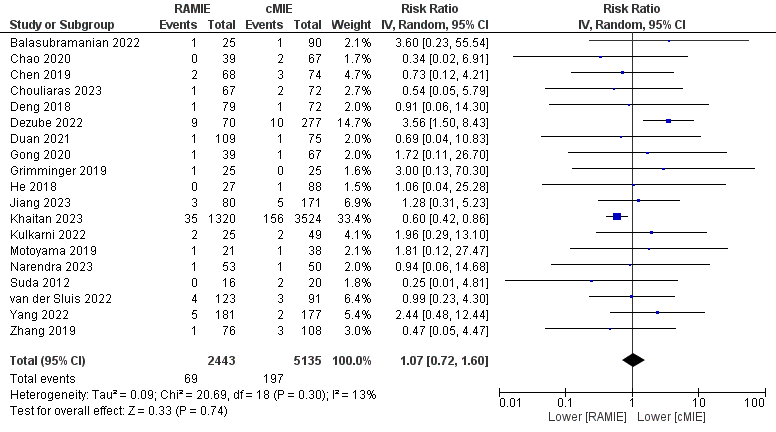


Supplementary Figure 19 Chyle Leakage


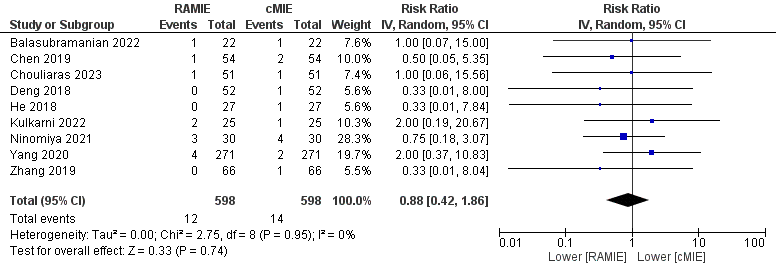


Supplementary Figure 20 Chyle Leakage, PSM


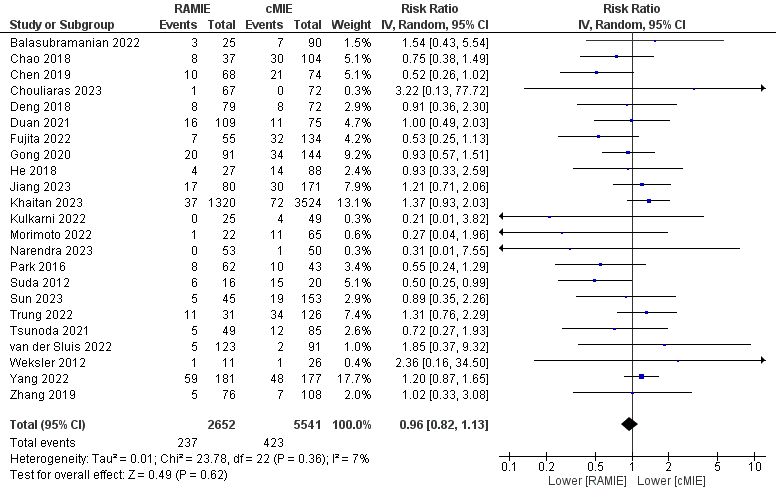


Supplementary Figure 21 RLN Palsy


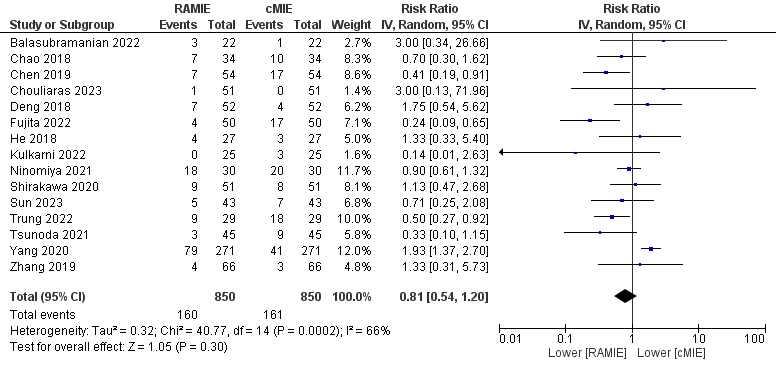


Supplementary Figure 22 RLN Palsy, PSM


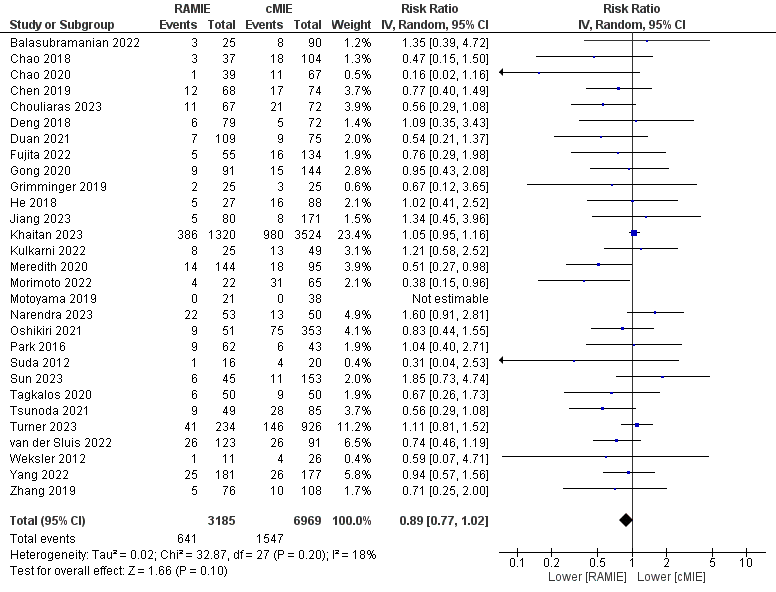


Supplementary Figure 23 Pulmonary Complications


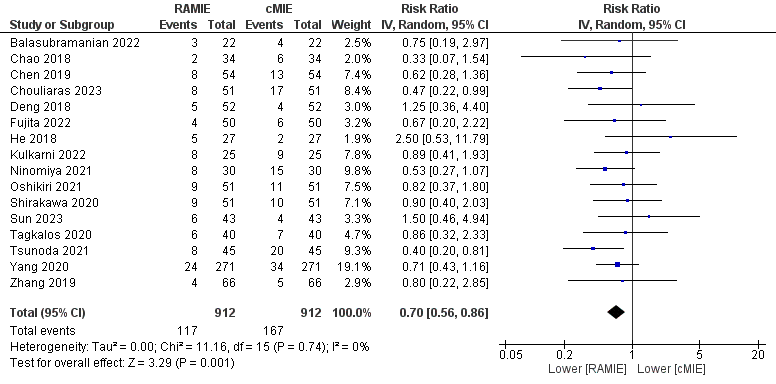


Supplementary Figure 24 Pulmonary Complications, PSM


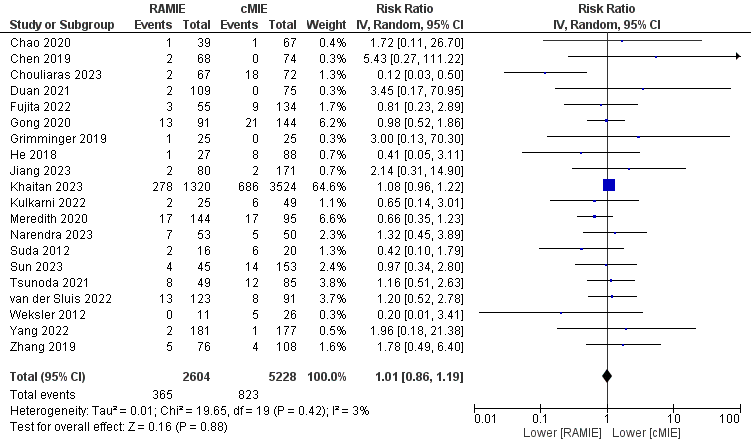


Supplementary Figure 25 Cardiac Complications


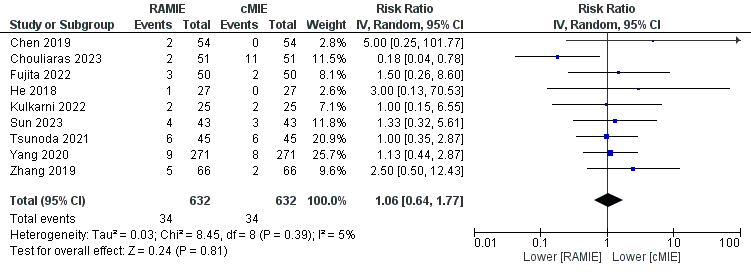


Supplementary Figure 26 Cardiac Complications, PSM


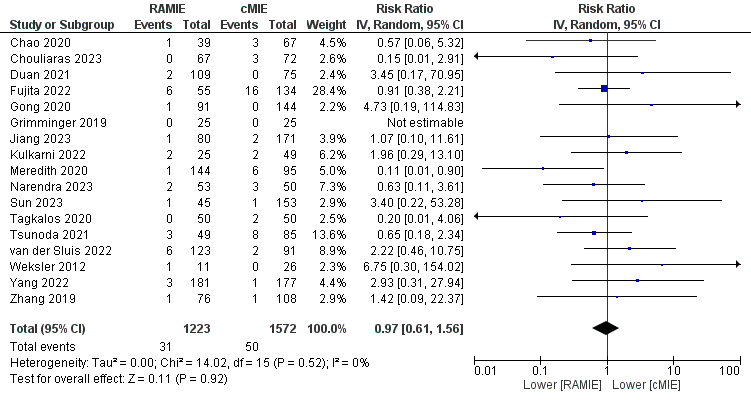


Supplementary Figure 27 Infectious Complications


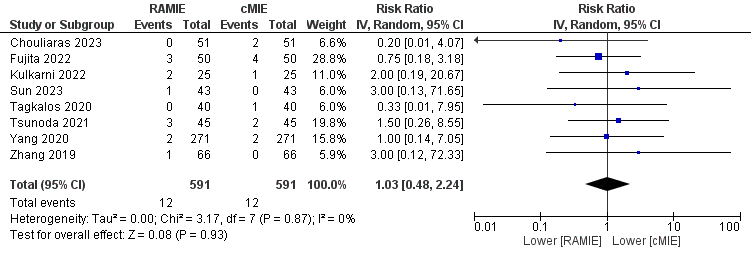


Supplementary Figure 28 Infectious Complications, PSM


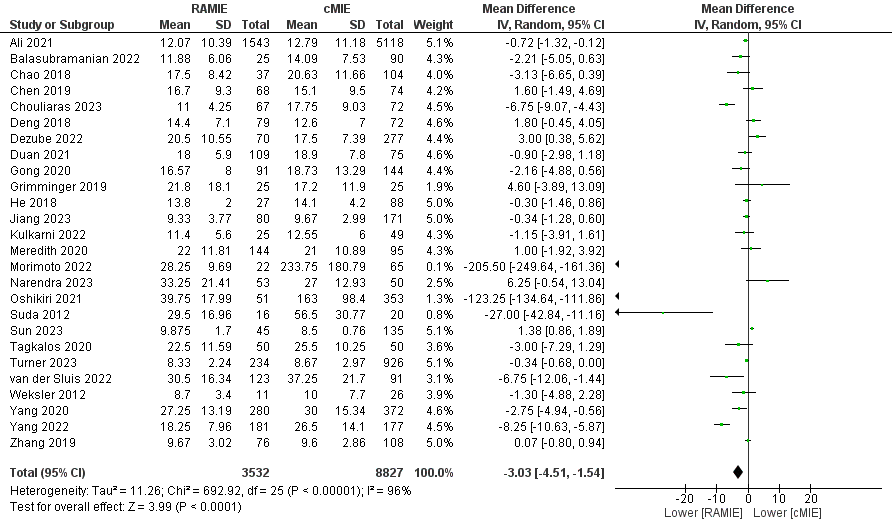


Supplementary Figure 29 Length of Hospital Stay


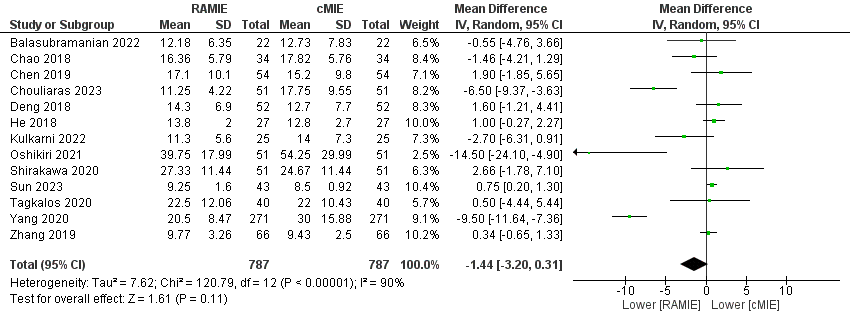


Supplementary Figure 30 Length of Hospital Stay, PSM


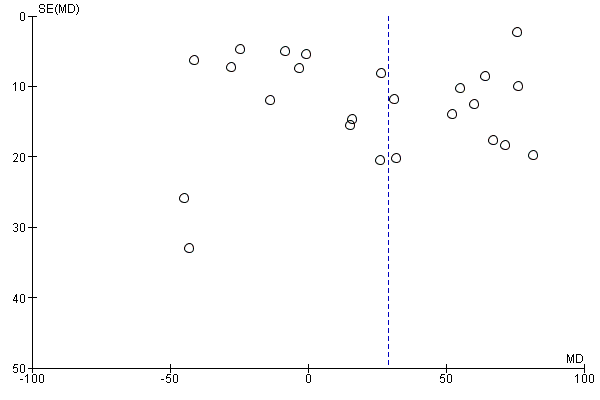


Supplementary Figure 31 Funnel Plot - Operating Time


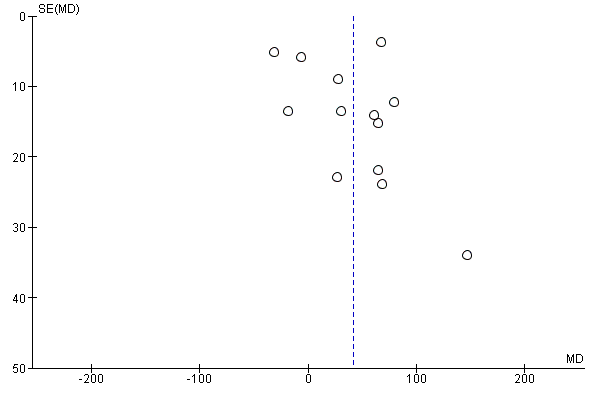


Supplementary Figure 32 Funnel Plot - Operating Time, PSM


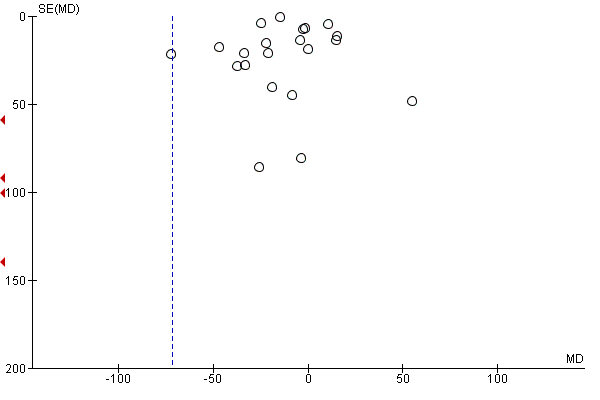


Supplementary Figure 33 Funnel Plot - Estimated Blood Loss


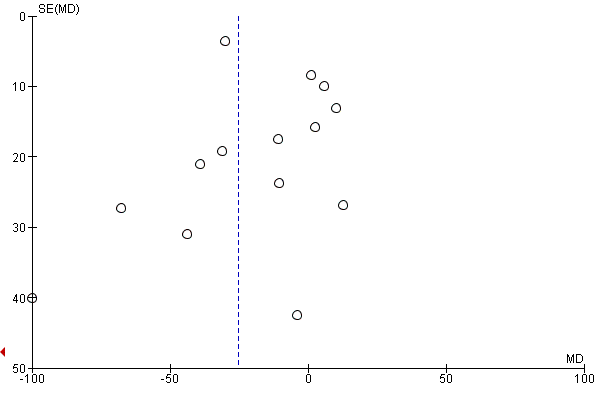


Supplementary Figure 34 Funnel Plot - Estimated Blood Loss, PSM


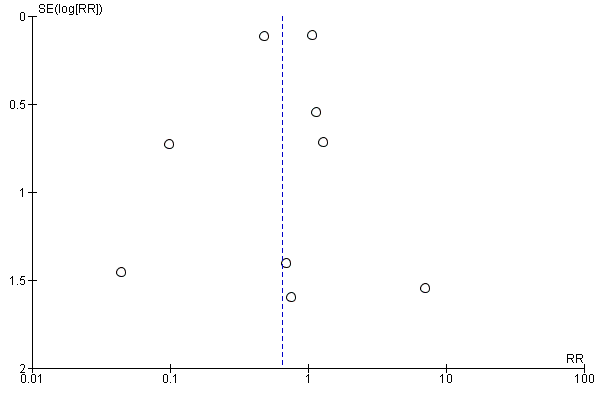


Supplementary Figure 35 Conversion to Open Procedure


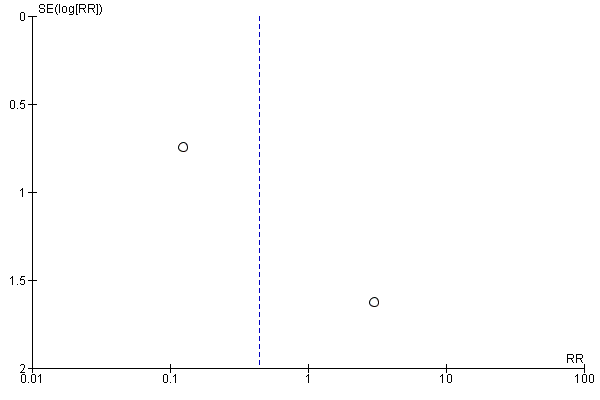


Supplementary Figure 36 Funnel Plot - Conversion to Open Procedure, PSM


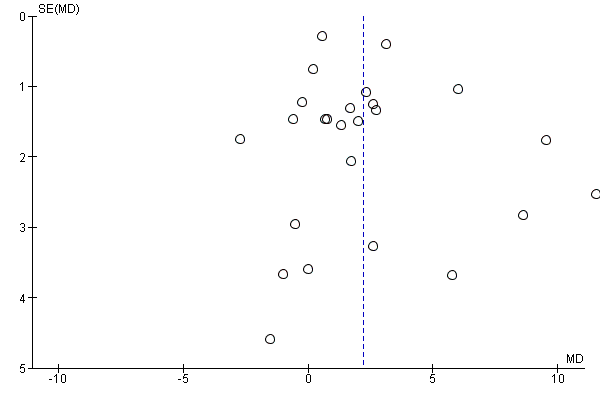


Supplementary Figure 37 Funnel Plot - Harvested Lymph Nodes, TOTAL


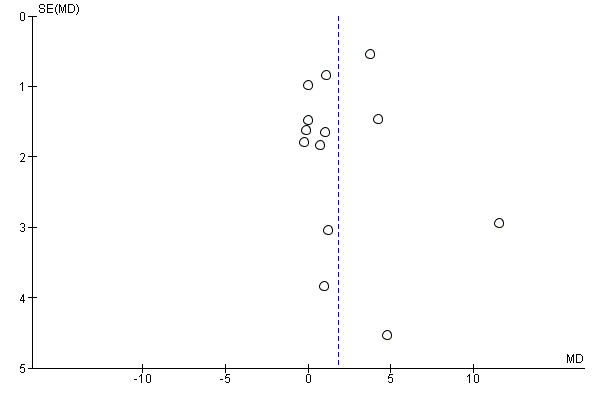


Supplementary Figure 38 Funnel Plot - Harvested Lymph Nodes, TOTAL, PSM


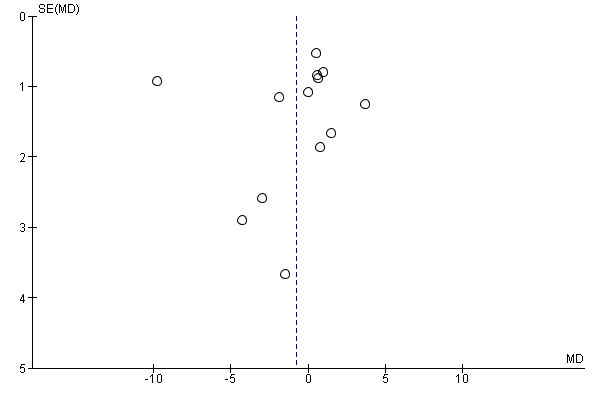


Supplementary Figure 39 Funnel Plot - Harvested Lymph Nodes, MEDIASTINAL


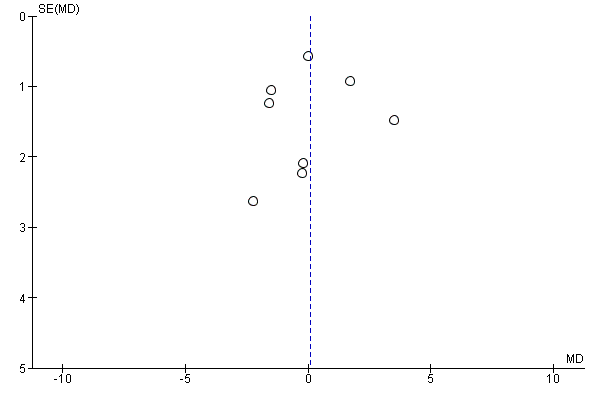


Supplementary Figure 40 Funnel Plot - Harvested Lymph Nodes, MEDIASTINAL, PSM


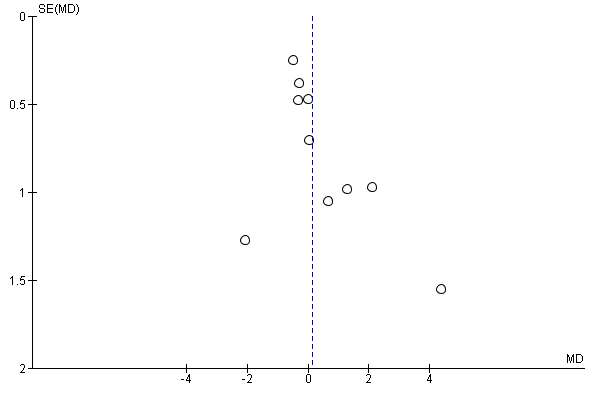


Supplementary Figure 41 Funnel Plot - Harvested Lymph Nodes, ABDOMINAL


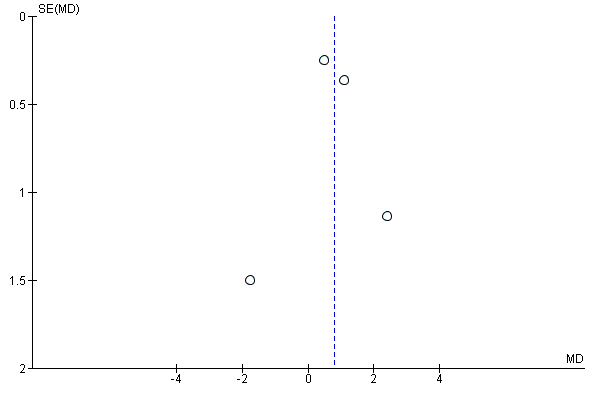


Supplementary Figure 42 Funnel Plot - Harvested Lymph Nodes, ABDOMINAL, PSM


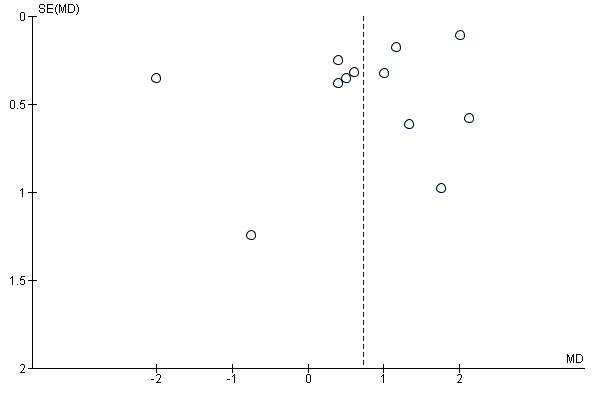


Supplementary Figure 43 Funnel Plot - Harvested Lymph Nodes, Left RLN


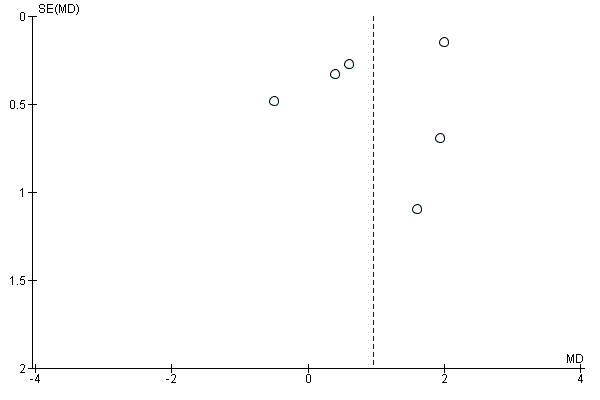


Supplementary Figure 44 Funnel Plot - Harvested Lymph Nodes, Left RLN, PSM


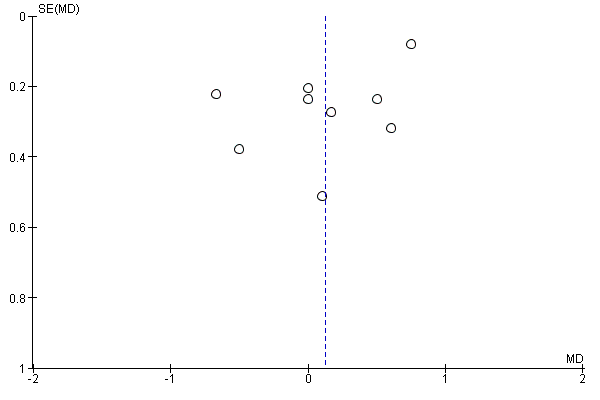


Supplementary Figure 45 Funnel Plot - Harvested Lymph Nodes, Right RLN


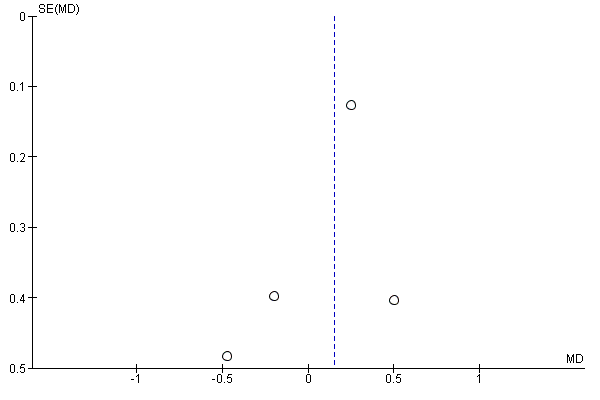


Supplementary Figure 46 Funnel Plot - Harvested Lymph Nodes, Right RLN, PSM


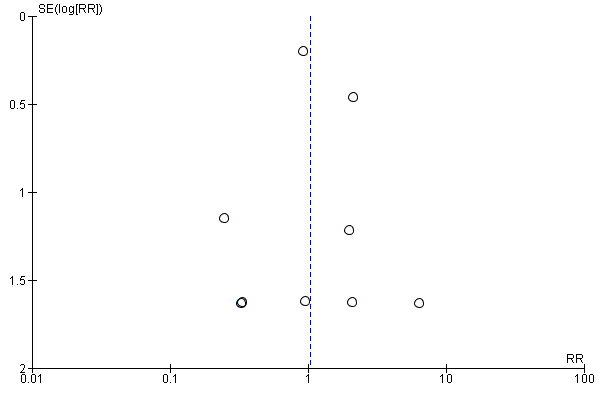


Supplementary Figure 47 Funnel Plot - 30-Day Mortality


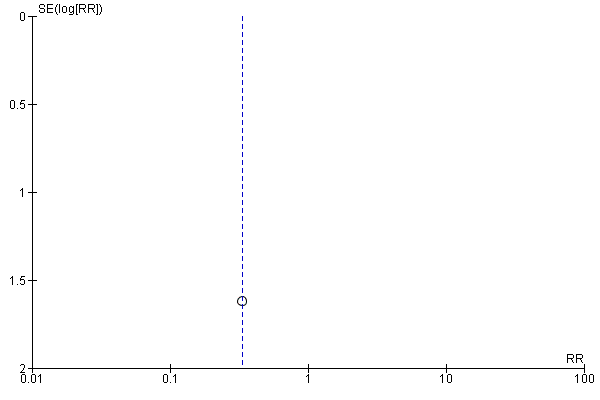


Supplementary Figure 48 Funnel Plot - 30-Day Mortality, PSM


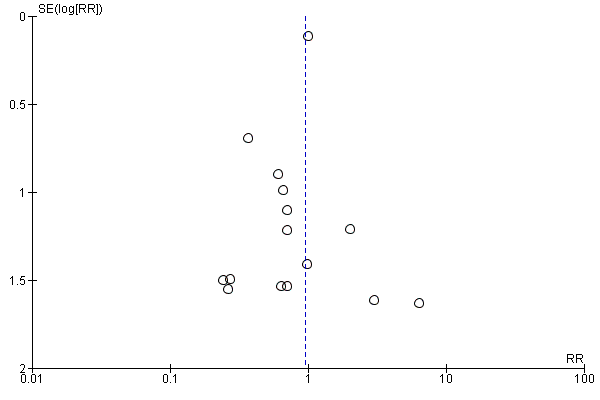


Supplementary Figure 49 Funnel Plot - 90-Day Mortality


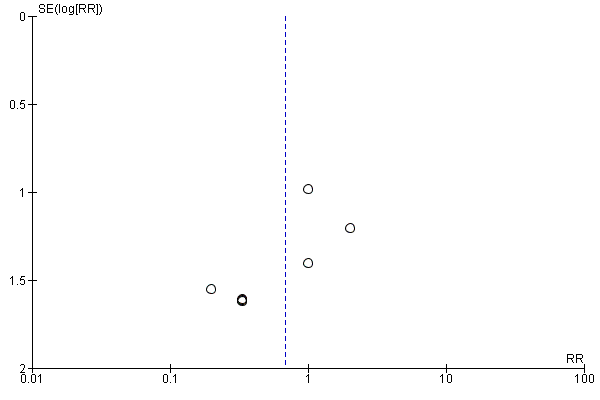


Supplementary Figure 50 Funnel Plot - 90-Day Mortality, PSM


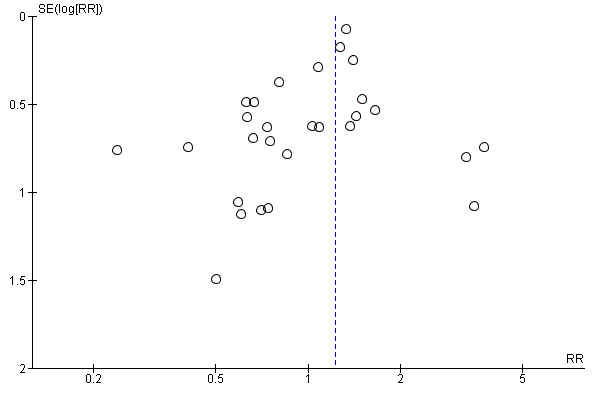


Supplementary Figure 51 Funnel Plot - Anastomotic Leakage


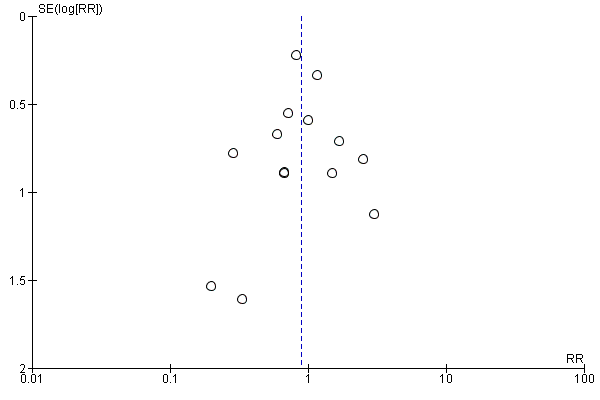


Supplementary Figure 52 Funnel Plot - Anastomotic Leakage, PSM


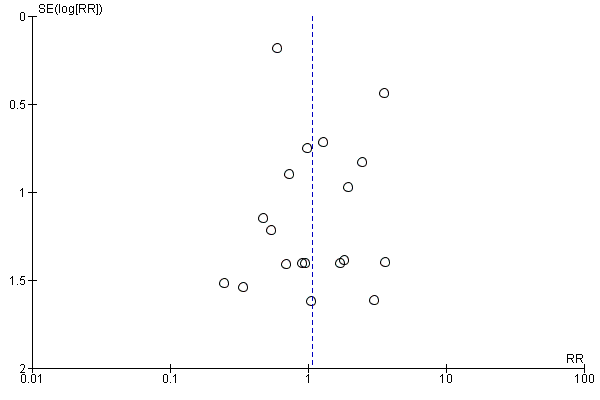


Supplementary Figure 53 Funnel Plot - Chyle Leakage


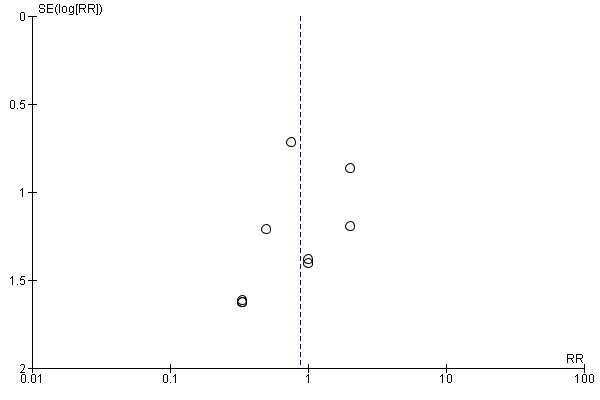


Supplementary Figure 54 Funnel Plot - Chyle Leakage, PSM


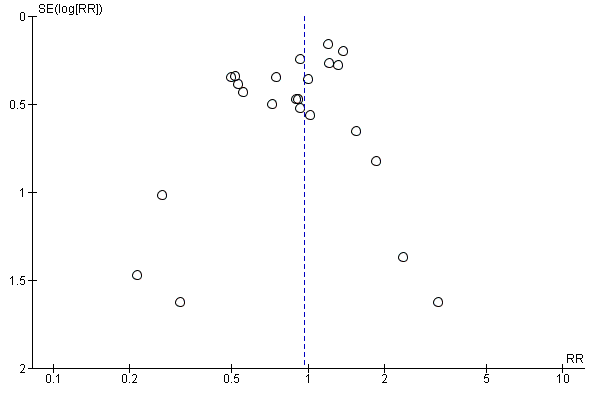


Supplementary Figure 55 Funnel Plot – RLN Palsy


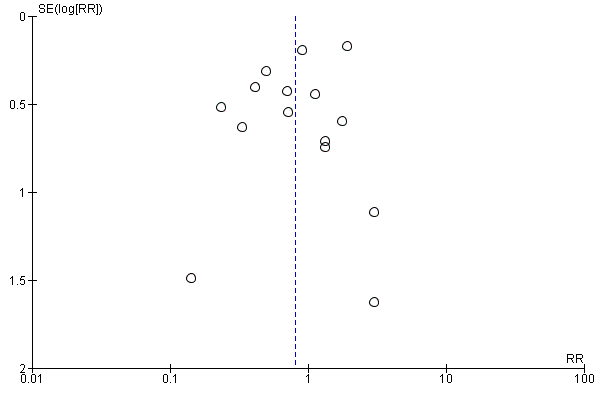


Supplementary Figure 56 Funnel Plot – RLN Palsy, PSM


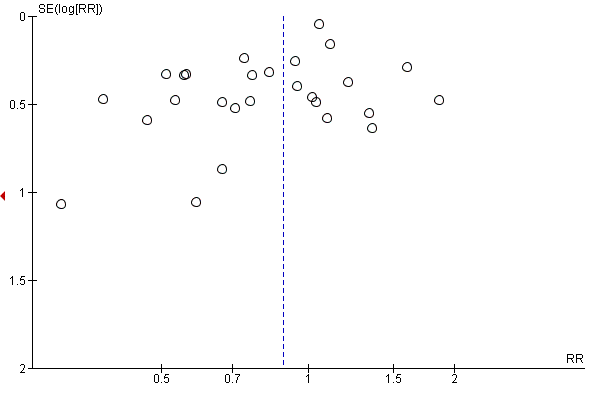


Supplementary Figure 57 Funnel Plot - Pulmonary Complications


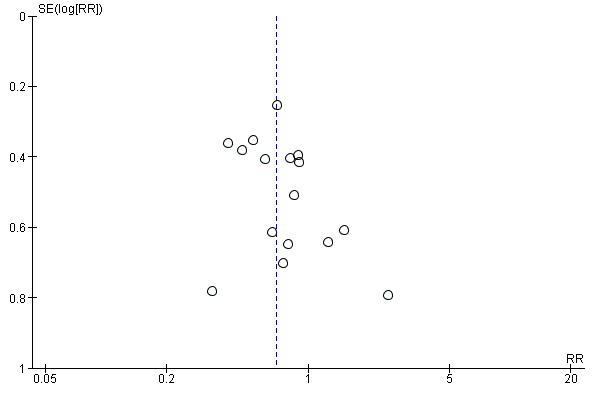


Supplementary Figure 58 Funnel Plot - Pulmonary Complications, PSM


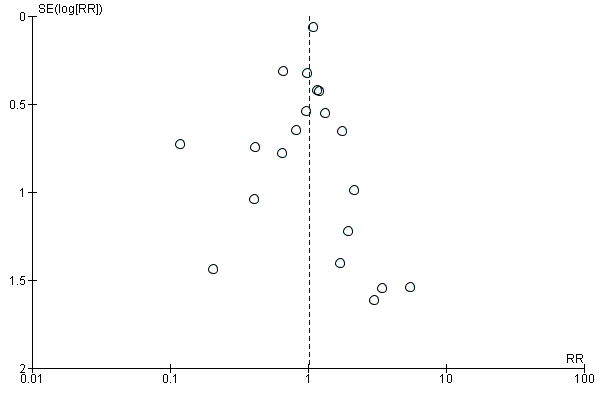


Supplementary Figure 59 Funnel Plot - Cardiac Complications


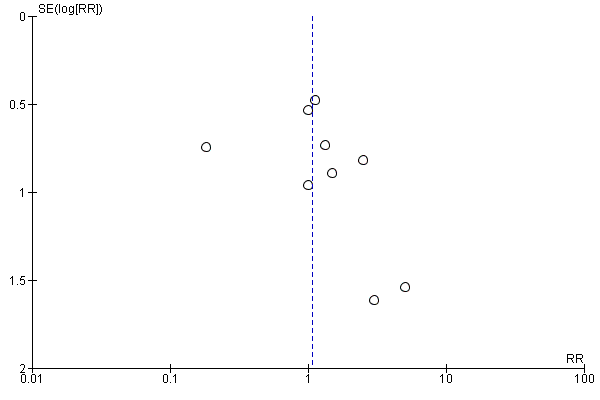


Supplementary Figure 60 Funnel Plot - Cardiac Complications, PSM


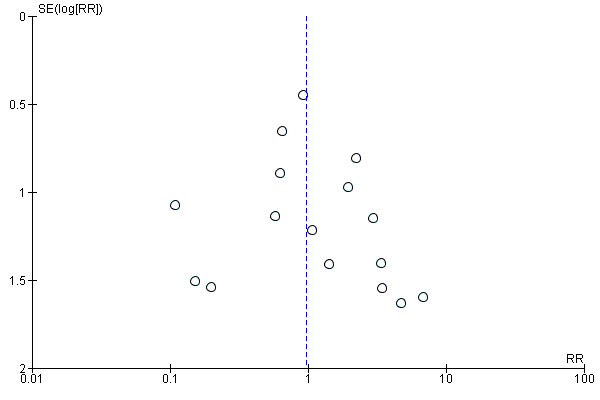


Supplementary Figure 61 Funnel Plot - Infectious Complications


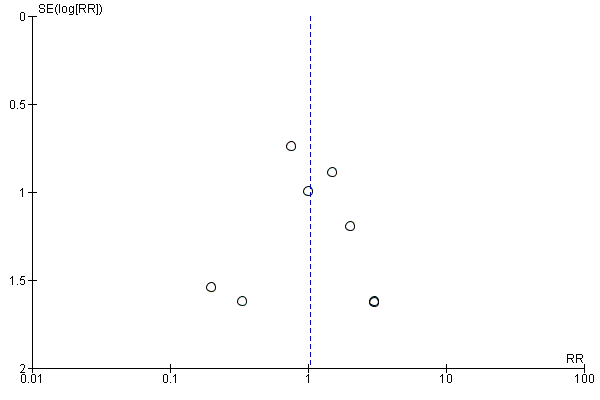


Supplementary Figure 62 Funnel Plot - Infectious Complications, PSM


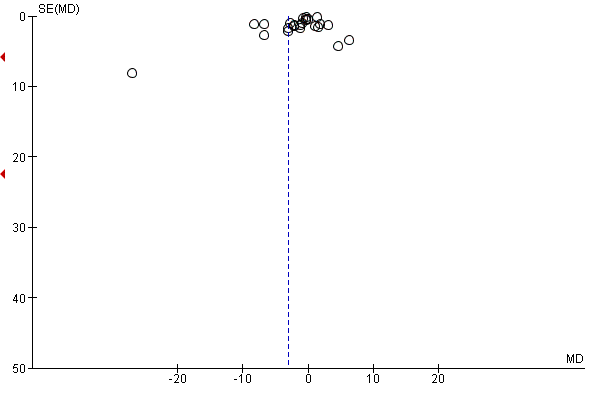


Supplementary Figure 63 Funnel Plot - Length of Hospital Stay


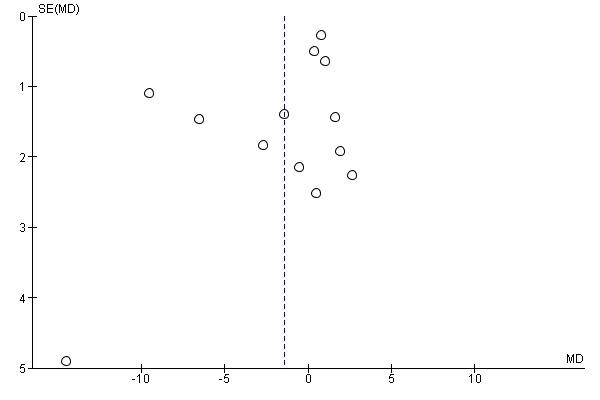


Supplementary Figure 64 Funnel Plot - Length of Hospital Stay, PSM
